# On the spore ornamentation of the microsoroid ferns (microsoroideae, polypodiaceae)

**DOI:** 10.1007/s10265-020-01238-4

**Published:** 2020-11-29

**Authors:** Chi-Chuan Chen, Ho-Yih Liu, Cheng-Wei Chen, Harald Schneider, Jaakko Hyvönen

**Affiliations:** 1 Organismal and Evolutionary Biology, Viikki Plant Science Center and Finnish Museum of Natural History (Botany), University of Helsinki, PO Box 7, 00100 Helsinki, FI Finland; 2 Department of Biological Sciences, National Sun Yat-Sen University, 70 Lien-hai Rd, Kaohsiung, 804 Taiwan; 3 Independent Researcher, Keelung, 202 Taiwan; 4 Center for Integrative Conservation, Xishuangbanna Tropical Botanical Garden, Chinese Academy of Sciences, Yunnan, China

**Keywords:** Ancestral state reconstruction, Palynology, Phylogeny, Scanning electron microscopy (SEM)

## Abstract

Microsoroideae is the third largest of the six subfamilies of Polypodiaceae, containing over 180 species. These ferns are widely distributed in the tropical and subtropical regions of the Old World and Oceania. We documented the spore ornamentation and integrated these data into the latest phylogenetic hypotheses, including a sampling of 100 taxa representing each of 17 major lineages of microsoroid ferns. This enabled us to reconstruct the ancestral states of the spore morphology. The results show verrucate ornamentation as an ancestral state for Goniophlebieae and Lecanoptereae, globular for Microsoreae, and rugulate surface for Lepisoreae. In addition, spore ornamentation can be used to distinguish certain clades of the microsoroid ferns. Among all five tribes, Lecanoptereae show most diversity in spore surface ornamentation.

**Electronic supplementary material:**

The online version of this article (10.1007/s10265-020-01238-4) contains supplementary material, which is available to authorized users.

## Introduction

The microsoroid ferns (Microsoroideae) are one of the largest subfamilies of Polypodiaceae, distributed mainly in the tropical and subtropical regions of the Old World. The generic classification of some genera nested in this lineage has been controversial, in particular the generic delimitation of *Leptochilus* Kaulf., *Microsorum* Link, and *Phymatosorus* Pic. Serm. that have been treated in previous taxonomic studies (e.g. Bosman 1991; Nooteboom 1997). The use of sequence-level data has further advanced studies on the phylogeny of the microsoroid ferns (Chen et al. 2020; Kreier et al. 2008; Testo et al. 2019; Wang et al. 2010; Zhang et al. 2019; Zhao et al. 2019). Based on the latest classification (Chen et al. 2020; PPG I 2016; Testo et al. 2019; Zhang et al. 2020), there are 16 currently accepted genera: *Bosmania* Testo, *Dendroconche* Copel., *Ellipinema* Li Bing Zhang and Liang Zhang, *Goniophlebium* (Blume) C. Presl, *Lecanopteris* Reinw. ex Blume, *Lemmaphyllum* C. Presl, *Lepidomicrosorium* Ching and K.H.Shing, *Lepisorus* (J.Sm.) Ching, *Leptochilus*, *Microsorum*, *Neocheiropteris* H. Christ, *Neolepisorus* Ching, *Paragramma* (Blume) T. Moore, *Thylacopteris* Kunze ex J. Sm., *Tricholepidium* Ching, and *Zealandia* Testo and A. R. Field. The number of genera may be reduced by expanding the definition of *Lepisorus* to also include *Ellipinema*, *Lemmaphyllum*, *Lepidomicrosorium*, *Neocheiropteris*, *Neolepisorus*, *Paragramma*, and *Tricholepidium* (Zhao et al. 2019). In total this group includes over 180 species but the species number may be underestimated in the species rich lineages such as *Goniophlebium*, *Leptochilus* and *Lepisorus* (Chen et al. 2020; PPG I 2016; Testo et al. 2019). In addition to the generic rank, authors have also proposed different ranks above and below genus, for example tribes (Chen et al. 2020), and subclades of the larger genera such as *Leptochilus* and *Lepisorus* (Wang et al. 2010; Zhang et al. 2019; Zhao et al. 2019). These latest studies have clarified such relationships, but there are still uncertainties that need further examination. For example, there seems to be an inconsistency in obtained results based on nuclear versus chloroplast genes (Nitta et al. 2018).

Spore morphology provides valuable information that helps to clarify taxonomy of the many lineages of ferns (Wagner 1974). Numerous studies using these characters have been carried out, such as the landmark publication by Tryon and Lugardon (1991) integrating information obtained using scanning (SEM) and transmission electron microscopy (TEM). Such studies have also been undertaken in Polypodiaceae focusing on morphological variation and sporogenesis (e.g., Giudice et al. 2004; Lloyd 1981; Morbelli and Giudice 2010; Van Uffelen and Hennipman 1985; Van Uffelen 1992,1993,1997; Wang 2001). Despite the limited taxon sampling in these studies, some general trends have been observed. For example, Tryon and Lugardon (1991) pointed out that the spore ornamentation of *Colysis ampla* Copel. (= *Dendroconche ampla* (F. Muell. ex Benth.) Testo, Sundue, and A.R. Field) differed from the other species assigned to *Colysis* C. Presl (= *Leptochilus*). Phylogenetic analyses showed this species to belong *Dendroconche*, and not to *Leptochilus* (Chen et al. 2020; Testo et al. 2019). In addition, spore ornamentation of the broadly defined *Phymatosorus* Pic.Serm has been found to be heterogenous (Tryon and Lugardon 1991), which is consistent with the polyphyly of the genus in the latest phylogenetic analyses (Chen et al. 2020).

Using the most recent robust phylogenetic hypotheses, it is now possible to re-evaluate the taxonomic value of the spore wall ornamentation. To achieve this, we integrated the spore surface data of the microsoroid ferns from previous, and our own studies, into the latest phylogenetic hypotheses with the aim to reconstruct the ancestral spore type for each clade/genus, as presented in Chen et al. (2020), and to assess possible trends of the spore surface development in the microsoroid ferns.

## Materials and methods

### Taxon sampling and the chloroplast DNA sequencing

Based on the latest hypothesis of phylogeny, and the available spore surface data, we chose species from each of the 15 out of 16 currently accepted genera, plus two *Microsorum* groups MG4 and MG5 (Chen et al. 2020; Testo et al. 2019; Zhang et al. 2020), with at least one species per genus/group, but recently described *Ellipinema* (Zhang et al. 2020) was not included because we did not have access to any material of this new genus. We also considered the subclades of larger genera, such as *Lepisorus*, and sampled them as thoroughly as possible (Wang et al. 2010; Zhao et al. 2019). In total, 98 out of 183 microsoroids species and two outgroup species, *Aglaomorpha meyeniana* Schott and *Pyrrosia polydactyla* (Hance) Ching, were included.

The chloroplast sequences (*rbcL*, *rps4* + *rps4-trnS*, *trnL* + *trnL-trnF*, *atpA*, *atpB* and *matK*) for molecular analyses were mostly those used in previous studies (e.g., Chen et al. 2020), but several previously unpublished sequences were added to the analyses here. Voucher information and Genbank accession numbers are provided in Table 1. DNA extraction, amplification, and sequencing methods are described in Chen et al. (2020).

**Table 1:** List of material used for obtaining sequences given as taxon name, voucher specimen with collecting locality, collector, or specimen number and the herbarium where deposited, followed by GenBank accession numbers for six plastid regions: *rbcL*, *rps4* and *rps4-trnS*, *trnL* and *trnL-trnF*, *atpA*, *atpB* and *rbcL-atpB*, *matK*. A dash indicates missing sequence. Sequences in bold are novel

Taxon	Voucher	rbcL	rps4-trnS	trnL-trnF	atpA	atpB	matK
*Bosmania*							
* Bosmania lastii* (Baker) Testo	Perier 7937 (P)	EU482961	EU483012	EU483058	–	–	–
* Bosmania membranacea* (D. Don) Testo	Taiwan: Taipei; CC.Chen 1077 (H)	MH051175	MH113474	MH113507	MH113541	MH113574	MH113607
*Dendroconche*							
* Dendroconche ampla* (F. Muell. ex Benth.) Testo, Sundue, and A.R. Field	Kessler 14,358 (VT)	KF570108	KF570109	KF570110	–	–	–
* Dendroconche linguiforme* (Mett.) Testo, Sundue, and A.R. Field	Solomon Islands; Wade 2887 (TAIF)	MH051174	MH113473	MH113506	MH113540	MH113573	MH113606
* Dendroconche scandens* (G. Forst.) Testo, Sundue, and A.R. Field	Australia: Victoria; CC.Chen 1080 (H)	MH051182	MH113481	MH113514	MH113547	MH113581	MH113614
*Goniophlebium*							
* Goniophlebium amoenum* (Wall. ex Mett.) Bedd	Cult. Xishuangbanna Bot. Gard. [Orig. Guangxi]; 00,2002,0891	MH665028	MH665091	MH665158	MH664988	MH665004	MH665018
* Goniophlebium argutum* (Wall. ex Hook.) J. Sm. ex Hook	Taiwan; Cranfill TW075 (UC)	DQ164442	DQ164473	DQ164505	–	–	–
* Goniophlebium chinense* (Christ) X.C. Zhang	Mainland China: Mt. Jinfo; Lu SG-X14 (PYU) Mainland China; Wei X.P. wxp201718 (IMD)	DQ078630 –	DQ078637 –	– –	– –	– –	– MF450478
* Goniophlebium formosanum* (Baker) Rodl-Linder	Taiwan; Cranfill TW043 (UC) Taiwan; Ranker 1998 (COLO)	– –	AY096224 –	DQ642235 –	– EF463813	– EF463495	– –
* Goniophlebium manmeiense* (Christ) Rodl-Linder	Mainland China: Lijiang; Lu SG-K4 (PYU)	DQ078628	DQ078631	–	–	–	–
* Goniophlebium mengtzeense* (Christ) Rodl-Linder	Mainland China; Barrington 2085a (UVM)	AY362560	AY362627	–	–	–	–
* Goniophlebium microrhizoma* (C.B. Clarke ex Baker) Bedd	Mainland China: Yunnan, Lijiang; Lu SG-K8 (PYU)	DQ078627	DQ078632	–	–	–	–
* Goniophlebium niponicum* (Mett.) Bedd. var. *niponicum*	Japan; Kato et al. (TI)	-	AY362626	EU483027	–	–	–
* Goniophlebium niponicum* var. *wattii* (Bedd.) Bedd	Mainland China: Yunnan, Kunming; Lu SG-D6 (PYU)	DQ078625	DQ078636	–	–	–	–
* Goniophlebium persicifolium* (Desv.) Bedd	Cult. Bot. Gard. Berlin-Dahlem; 239–12-90–33 (B)	EU482933	EU482978	EU483028	–	–	–
* Goniophlebium pseudoconnatum* (Copel.) Copel	cult. Bot. Gard. Berlin-Dahlem; 239–36-90–30 (B)	EU482934	EU482979	EU483029	–	–	–
* Goniophlebium subauriculatum* (Blume) C.Presl	Cult. Uni. California Bot. Gard. [Orig. Java, (UC)] cult. Bot. Gard. Göttingen, Kreier s.n. (GOET)	AF470342 –	– DQ168812	AY083645 –	– –	– –	– –
***Lecanopteri***							
* Lecanopteris carnosa* (Reinw.) Blume	Cult. Utrecht Bot. Gard. [Orig. Sulawesi; David Klein s.n. (L)] Cult. Bot. Gard. Kew; Cranfill 153 (UC)	AF470322 –	– AY096227	AY083625 –	– –	– –	– –
* Lecanopteris celebica* Hennipman	Cult. UBG 85GR00170 [Sulawesi Island; Hennipman s.n. (L)] Cult. Bot. Gard. Göttingen; Schneider s.n. (GOET)	AF470323 –	– EU482981	AY083626 –	– –	– –	– –
* Lecanopteris mirabilis* (C. Chr.) Copel	Cult. Utrecht Bot. Gard. 665 [Orig. New Guinea; Hennipman s.n. (U)]	AF470330	EU482984	AY083633	–	–	–
* Lecanopteris sinuosa* (Hook.) Copel	Cult. Utrecht Bot. Gard. 87GR00087 [Philippine; Hennipman 7821 (U, L)] Australia; Sankowsky 4169 (NSW)	AF470321 –	AY362634 –	AY083624 –	– KP164484	– KP164491	– –
*Lemmaphyllum*							
* Lemmaphyllum carnosum* (Wall. ex J. Sm.) C. Presl	Japan; Zhang 4364 (PE)	GU126698	GU126717	GU126728	–	GU126706	-
* Lemmaphyllum drymoglossoides* (Baker) Ching	Mainland China: Guangxi; Wei XP et al. wxp117 (PE)	KX891372	KX891403	KX891357	–	KX891385	-
* Lemmaphyllum microphyllum* C. Presl	Cult. Bot. Gard. Zurich; Schneider s.n. (GOET) Taiwan: Ilan; Ranker 2010 (COLO)	EU482938 –	EU482988 –	EU483033 –	- EF463824	– EF463496	– –
* Lemmaphyllum rostratum* (Bedd.) Tagawa	Hainan Island; Wei XP et al. wxp108 (PE)	KX891376	KX891407	KX891363	–	KX891390	MF450477
*Lepidomicrosorium*							
* Lepidomicrosorium buergerianum* (Miq.) Ching and K.H. Shing	Mainland China: Yunnan; Shui 80,894 (PE)	GQ256315	GQ256392	GQ256242	–	GQ256156	–
* Lepidomicrosorium superficiale* (Blume) L. Wang	Taiwan: Ilan; CC.Chen 1104 (HITBC)	MH051159	MH113458	MH113492	MH113525	MH113558	MH113591
***Lepisorus***							
* Lepisorus accedens* (Blume) Hosok	East Kalimatan; Hovenkamp 05–298 (L) Philippines; Philippines233	EU482936 –	EU482986 –	EU483031 –	–	–KX891383	–
* Lepisorus affinis* Ching	Cult. Fairylake Bot. Gard.; Zhang 4219 (PE)	GQ256256	GQ256328	GQ256173	–	GQ256086	–
* Lepisorus angustus* Ching	Tibetan Plateau; Shen Z.H. S25 (PE)	GQ256290	GQ256364	GQ256214	–	GQ256127	-
* Lepisorus annuifrons* (Makino) Ching	Japan; Kyoto Kokubo s.n. (TI)	GQ256258	GQ256331	GQ256176	–	GQ256089	-
* Lepisorus asterolepis* (Baker) Ching ex S.X. Xu	Mainland China: Sichuan; Zhang 5171 (PE)	GQ256259	GQ256332	GQ256177	–	GQ256090	–
* Lepisorus boninensis* (Christ) Ching	Cult. Tuebingen Bot. Gard. acc.54022 [Orig. Japan]	GQ256262	GQ256335	GQ256180	–	GQ256093	–
* Lepisorus clathratus* (C.B.Clarke) Ching	Mainland China: Beijing; jingB-1 (PE)	KY419704	KY419704	KY419704	KY419704	KY419704	KY419704
* Lepisorus contortus* (Christ) Ching	Mainland China: Chongqing; Zhang 5204 (PE)	GQ256265	GQ256338	GQ256183	–	GQ256096	–
* Lepisorus kawakamii* (Hayata) Tagawa	Taiwan; Ranker 2051 (COLO)	EU482940	EU482990	GQ256193	–	GQ256106	–
* Lepisorus kuchenensis* (Y.C. Wu) Ching	Mainland China: Guangxi; J.M. Xi 08,188 (PE)	GQ256272	GQ256346	GQ256194	–	GQ256107	–
* Lepisorus loriformis* (Wall. ex Mett.) Ching	Mainland China: Yunnan; C.D. Xu A0303 (PE)	GQ256313	GQ256389	GQ256240	–	GQ256153	–
* Lepisorus macrosphaerus* (Baker) Ching	Cult. Kunming Bot. Gard.; Kim 2012–3 (KUN)	JX103697	JX103739	JX103781	–	JX103655	–
* Lepisorus marginatus* Ching	Mainland China: Hubei; Zhang 3360 (PE)	GQ256281	GQ256355	GQ256204	–	GQ256117	–
* Lepisorus megasorus* (C. Chr.) Ching	Taiwan; Cranfill TW069 (UC)	DQ642158	DQ642196	DQ642240	–	GQ256119	–
* Lepisorus miyoshianus* (Makino) Fraser-Jenk. and Subh. Chandra	Mainland China: Sichuan; C.C. Liu DB06104 (PE) Taiwan; E. Schuettpelz 1136A (DUKE)	GQ256255 –	GQ256327 –	GQ256172 –	– KF909068	GQ256085 –	– KF909023
* Lepisorus morrisonensis* (Hayata) H. Itô	Tibetan Plateau; Zhang 5113 (PE)	GQ256284	GQ256358	GQ256208	–	GQ256121	–
* Lepisorus mucronatus* (Fée) Li Wang	Malaysia; Jaman 5891 (UC)	AY362562	AY362629	GQ256168	–	GQ256081	–
* Lepisorus obscurevenulosus* (Hayata) Ching	Mainland China: Guangxi; Zhang 4151 (PE)	GQ256286	GQ256360	GQ256210	–	GQ256123	–
* Lepisorus oligolepidus* (Baker) Ching	Tibetan Plateau; Zhang 5082 (PE)	GQ256287	GQ256361	GQ256211	–	GQ256124	–
* Lepisorus onoei* (Franch. and Sav.) Ching	Japan; Zhang 4352 (PE)	GQ256288	GQ256362	GQ256212	–	GQ256125	–
* Lepisorus platyrhynchos* (Kunze) Li Wang	Cult. Bot. Gard. Zurich; Kreier s.n. (GOET)	DQ642152	DQ642190	DQ642233	–	GQ256082	–
* Lepisorus pseudonudus* Ching	Mainland China: Sichuan; Zhang 4249 (PE)	GQ256291	GQ256365	GQ256215	–	GQ256128	–
* Lepisorus pseudoussuriensis* Tagawa	Taiwan; Cranfill TW093 (UC)	EU482943	EU482993	GQ256216	–	GQ256129	–
* Lepisorus rotundus* Ching	Tanzania: Kilimanjaro; RV 7675	HQ711996	HQ712012	HQ712015	–	HQ712006	–
* Lepisorus scolopendrium* (Ching) Mehra and Bir	Laos; Wu 2441 (KUN)	JX103698	JX103740	JX103782	–	JX103656	–
* Lepisorus spicatus* (L.f.) Li Wang	Cult. Bot. Gard. Goettingen; Schneider s.n. (GOET) Tahiti; Ranker 1915 (COLO)	DQ642153 -	DQ642191 -	DQ642234 -	– EF463800	– EF463490	– –
* Lepisorus sublinearis* (Baker ex Takeda) Ching	Mainland China: Yunnan; Shui 80,595/81,060 (PE)	GQ256301	GQ256375	GQ256226	–	GQ256138	–
* Lepisorus thunbergianus* (Kaulf.) Ching	Mainland China: Anhui, Huangshan; CC.Chen 1064 (H)	**MT137054**	**MT137057**	**MT137059**	**MT137061**	**MT137062**	**MT137063**
* Lepisorus ussuriensis* (Regel and Maack) Ching	Mainland China: Heilongjiang; B.D. Liu s.n. (PE)	GQ256311	GQ256387	GQ256238	–	GQ256151	-
* Lepisorus soulieanus* (Christ) Ching and S.K. Wu	Mainland China: Sichuan; Zhang 5168 (PE)	GQ256321	GQ256399	GQ256249	–	GQ256163	–
* Lepisorus waltonii* (Ching) S.L. Yu	Tibetan Plateau; Zhang 4639 (PE)	GQ256322	GQ256400	GQ256250	–	GQ256164	–
*Leptochilus*							
* Leptochilus axillaris* (Cav.) Kaulf	Laos; Wu 2439 (KUN)	JX103700	JX103742	JX103784	–	JX103658	–
* Leptochilus cantoniensis* (Baker) Ching	Hainan Island; Kuo 1701 (TAIF)	**MT137055**	MH665095	MH665162	–	–	–
* Leptochilus decurrens* Blume	cult. Kunming Bot. Gard.; Kim 2012–12 (KUN)	JX103724	JX103766	JX103808	–	JX103682	–
* Leptochilus digitatus* (Baker) Noot	cult. Xishuangbanna Bot. Gard.; CC.Chen 1067 (H)	MH051162	MH113461	MH113495	MH113528	MH113561	MH113594
* Leptochilus ellipticus* (Thunb. ex Murray) Noot	Japan; Wade 3656 (TAIF)	MH665037	MH665101	MH665168	–	–	–
* Leptochilus ellipticus* var. *flexilobus* (Christ) X.C. Zhang	Mainland China: Hunan; R.H. Jiang	**MT137056**	**MT137058**	**MT137060**	**-**	**-**	**MT137064**
* Leptochilus ellipticu*s var. *pentaphyllus* (Baker) X.C. Zhang and Noot	Mainland China: Yunnan; Xu A0357 (PE)	MH665043	MH665108	MH665175	–	–	–
* Leptochilus hemionitideus* (C. Presl) Noot	cult. Xishuangbanna Bot. Gard.; CC.Chen 1066 (H)	MH051165	MH113464	MH175521	MH113531	MH113564	MH113597
* Leptochilus* x *hemitomus* (Hance) Noot	Mainland China; Zhang 3302 (PE)	EU482951	EU483001	EU483047	–	–	–
* Leptochilus henryi* (Baker) X.C. Zhang	Mainland China: Sichuan; Zhang 2541 (PE)	GQ256254	GQ256326	GQ256171	–	GQ256084	–
* Leptochilus heterophyllus* (S.K. Wu and K.L. Phan) Christenh	Vietnam; WP-201 (KUN)	JX520934	JX520936	JX520938	–	JX520932	–
* Leptochilus leveillei* (Christ) X.C. Zhang and Noot	Mainland China: Sichuan, Mt. Emei; SG Lu-EM26 (PYU)	EU363240	EU363254	-	–	–	–
* Leptochilus macrophyllus* (Blume) Noot	Indonesia: Java; Wade 1962 (TAIF)	MH051167	MH113466	MH113499	MH113533	MH113566	MH113599
* Leptochilus pedunculatus* (Hook. and Grev.) Fraser-Jenk	Vietnam: bugiamap; Wade 1334 (TAIF)	MH051168	MH113467	MH113500	MH113534	MH113567	MH113600
* Leptochilus pothifolius* (Buch.-Ham. ex D. Don) Fraser-Jenk	Taiwan; CC.Chen 1017 (H)	MH051163	MH113462	MH113496	MH113529	MH113562	MH113595
* Leptochilus pteropus* (Blume) Fraser-Jenk	Taiwan; CC.Chen 1010 (H)	MH051176	MH113475	MH113508	MH113542	MH113575	MH113608
* Leptochilus wrightii* (Hook. and Baker) X.C. Zhang	Taiwan: Kaohsiung; CC.Chen 1087 (H)	MH051170	MH113469	MH113502	MH113536	MH113569	MH113602
MG4 and MG5 (*M. commutatum and M. cuspidatum clades*)							
* Microsorum commutatum* (Bl.) Copel	Philippines; Wade 3768 (TAIF)	MH051171	MH113470	MH113503	MH113537	MH113570	MH113603
* Microsorum cuspidatum* (D. Don) Tagawa	Cult. Kunming Bot. Gard.; Kim 2012–6 (KUN)	JX103707	JX103749	JX103791	-	JX103665	-
* Microsorum hainanense* Noot	Cult. SCIB; Wang 1348 (PE)	EU482960	EU483011	EU483057	-	-	-
* Microsorum insigne* (Blume) Copel	Cult. Xishuangbanna Bot. Gard.; CC.Chen 1073 (H)	MH051172	MH113471	MH113504	MH113538	MH113571	MH113604
* Microsorum membranifolium* (R. Br.) Ching	Solomon Islands; Wade 2753 (TAIF)	MH665077	MH665143	MH665209	MH664996	MH665011	MH665023
* Microsorum rubidum* (Kunze) Copel	Taiwan: Pingtung; CC.Chen 1008 (H)	MH665085	MH665151	MH665215	MH665001	MH665015	MH665026
*Microsorum*							
* Microsorum glossophyllum* Copel	Solomon Islands; Wade 3053 (TAIF)	MH051180	MH113479	MH113512	MH175522	MH113579	MH113612
* Microsorum musifolium* (Blume) Copel	Cult. Dr. Cecilia Koo Bot. Cons. Center K013966 (H)	MH665080	MH665146	MH665212	MH664999	**MT157262**	-
* Microsorum punctatum* (L.) Copel	Taiwan: Pingtung; CC.Chen 1076 (H)	MH051178	MH113477	MH113510	MH113544	MH113577	MH113610
* Microsorum scolopendria* (Burm. f.) Copel	Taiwan; CC.Chen 1085 (H)	MH051190	MH113489	MH113522	MH113555	MH113588	MH113622
* Microsorum steerei* (Harr.) Ching	Taiwan; CC.Chen 1013 (H)	MH051183	MH113482	MH113515	MH113548	MH113582	MH113615
* Microsorum thailandicum* T. Booknerd and Noot	Cult. Bot. Gard. Göttingen; Schwertfeger s.n. (GOET)	EU482969	EU483020	EU483066	–	–	–
***Neocheiropteris***							
* Neocheiropteris palmatopedata* (Baker) Christ	Mainland China: Sichuan; Kuo 1552 (TAIF)	MH051185	MH113484	MH113517	MH113550	MH113584	MH113617
***Neolepisorus***							
* Neolepisorus ensatus* (Thunb.) Ching	Taiwan; CC.Chen 1011 (H)	MH051184	MH113483	MH113516	MH113549	MH113583	MH113616
* Neolepisorus fortunei* (T.Moore) Li Wang	Taiwan: Miaoli; CC.Chen 1012 (H)	MH051186	MH113485	MH113518	MH113551	MH113585	MH113618
* Neolepisorus ovatus* (Wall. ex Bedd.) Ching	Mainland China: Hubei; CC.Chen 1041 (H)	MH051187	MH113486	MH113519	MH113552	MH113586	MH113619
* Neolepisorus zippelii* (Blume) L. Wang	Indonesia: Java-Gede-Pangrango National Park; Wade 1794 (TAIF)	MH051188	MH113487	MH113520	MH113553	MH175523	MH113620
***Paragramma***							
* Paragramma longifolia* (Blume) T. Moore	Malay Peninsula; Cranfill BF012 (UC) cult. Bot. Gard. Munich-Nymphenburg; Schneider s.n. (GOET)	DQ642157 –	DQ642195 –	DQ642239 –	– EF463825	– EF463497	– –
***Thylacopteris***							
*Thylacopteris papillosa* (Blume) J.Sm	Borneo; Daniele Cicuzza 2258 (UBDH)	MH665089	MH665156	MH665220	MH665002	MH665016	-
***Tricholepidium***							
* Tricholepidium normale* (D. Don) Ching	Vietnam: Mt. Bidoup; Wade 2649 (TAIF)	MH175520	MH113490	MH113523	MH113556	MH113589	MH113623
* Zealandia*							
* Zealandia novae-zealandiae* (Baker) TestoandA. R. Field	New Zealand: Thames; Perrie et al. (WELT P20873)	DQ401116	DQ401126	DQ401121	–	–	–
* Zealandia pustulata* (G. Forst.) TestoandA. R. Field	Australia: Victoria; CC.Chen 1081 (H)	MH051181	MH113480	MH113513	MH113546	MH113580	MH113613
* Zealandia powellii* (Baker) Testo and A. R. Field	Solomon Islands; Wade 3352 (TAIF)	MH665081	MH665147	MH665213	MH665000	MH665014	MH665025
Outgroups							
* Aglaomorpha meyeniana* Schott	Cult. Goettingen; Janssen 2260 (GOET) Cult. Goettingen; Janssen V-17 (GOET) Cult. Dr. Cecilia Koo Bot. Cons. Center K016952	AY529153 – –	– AY459185 –	– – FJ807657	– – JF304020	– – –	– – JF303958
* Pyrrosia polydactyla* (Hance) Ching	Taiwan; Ranker 2080 (COLO) Taiwan; Knapp 3801 (P) Taiwan; Lu PF 21,430 (PE)	EF463259 – –	– KY931286 –	– KY931410 –	EF463844 – –	EF463511 – –	– – KY633008

### The spore data

Spore data were compiled by incorporating the results of previously published studies (Bosman 1991; Dai et al. 2006; Devi 1981; Hennipman 1990; Huang 1981; Jiang et al. 2010; Kholia et al. 2012; Large and Braggins 1991; Large et al. 1992; Mitui 1971,1977; Nayar and Devi 1964; Nooteboom 1997; Pal and Pal 1970; Qi and Zhang 2009; Rödl-Linder 1990,1994; Shalimov et al. 2013; Shi 2002; Shi and Zhang 1998; Sugong et al. 2005; Tryon and Lugardon 1991; van Uffelen 1993; Wang 2001; Zhang et al. 2006; Zink 1993) and novel observations partially based on the MSc thesis of the first author (Chen 2011). Spore samples were obtained from specimens recently collected in Taiwan, and from herbarium specimens of the National Sun Yat-Sen University of Taiwan (SYSU), and Taiwan Forestry Research Institute (TAIF). The new collected specimens were preserved as vouchers and deposited mainly in the SYSU (Table S1).

Spore surface ornamentation was observed and the size was measured using both light microscopy (LM) and SEM. For the size measurements, 10–20 untreated spores per accession were chosen randomly and measured using the program ImageJ (Schneider et al. 2012). The perispore was included in the measurements, and the data of spore size was described including both polar and equatorial diameter. For studies of the ornamentation, untreated spores were fixed on aluminum stubs, coated with ca. 15 nm of gold with the ion sputter (Hitachi E–101), and examined using SEM (Hitachi S2400 and TM3000) at 12–18 kV. Spores treated in this way remain suitable for examination with the SEM for at least one month (Van Uffelen and Hennipman 1985). Magnification of 1000–3000 X was used for the micrographs of the whole spores and 4000–8000 X for the surface details.

To integrate the spore ornamentation data, we chose to use the most common descriptions if there was conflict between published studies. The main spore surface ornamentation types were illustrated using the software Gimp (gimp.org).

### Terminology

We compared our observations with previously reported descriptions and images using the established descriptive terminology (Lellinger 2002; Punt et al. 2007; Shalimov et al. 2013; Tryon and Lugardon 1991). We studied and compiled data of two spore features: surface ornamentation and type of projections. Distinction of exospore and perispore requires more precise estimates using transmission electron microscopy (TEM). Numerous studies have tried to understand the spore wall structure of Polypodiaceae (e.g., Hennipman 1990; Tryon and Lugardon 1991; van Uffelen 1993), but the TEM data is still insufficient and thus, in this study, we treat the visible surface ornamentation as one character.

**Fig. 1:**
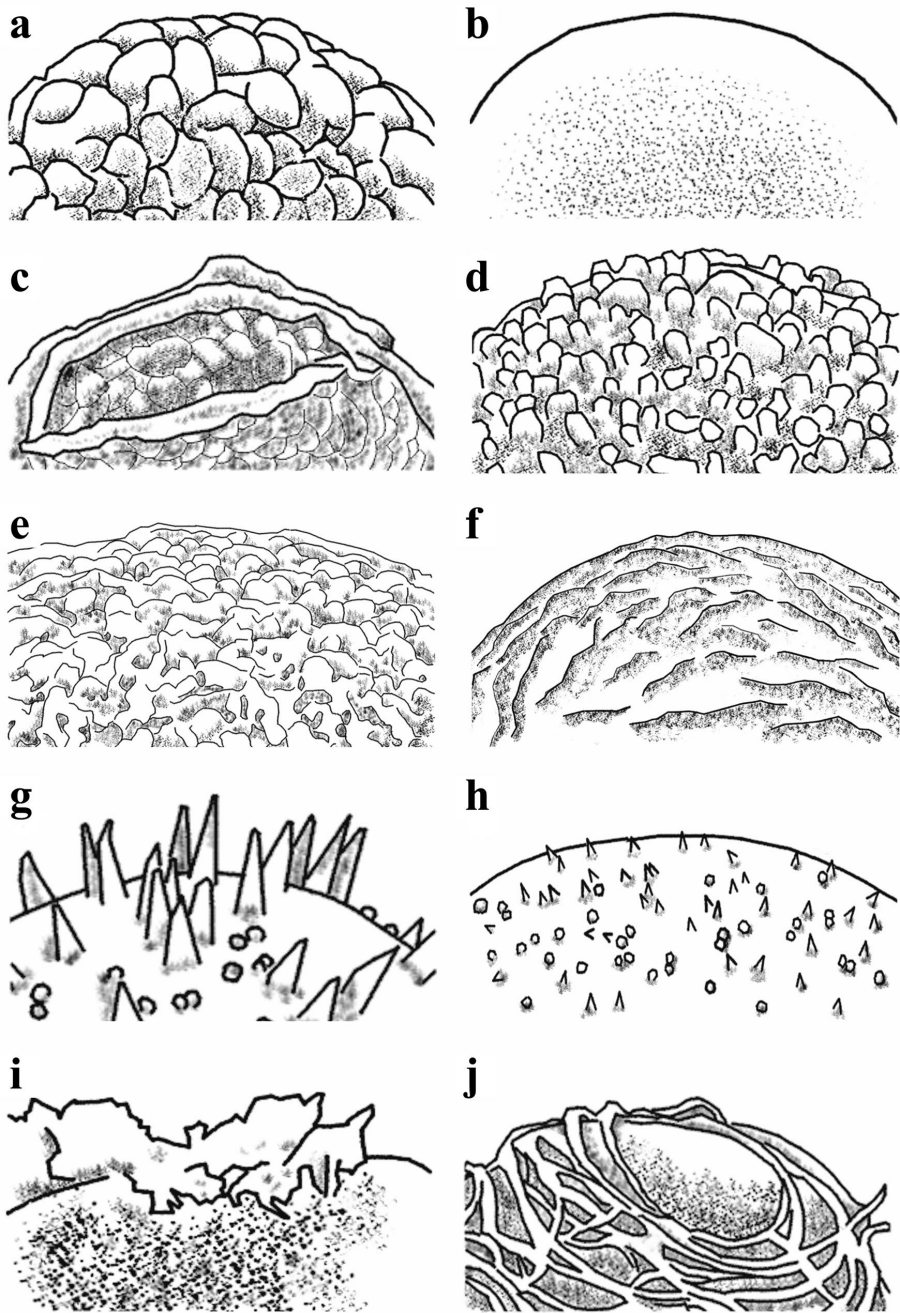
aMain spore surface ornamentation types used in this study. **a**, verrucate; (**b**), psilate; (**c**), verrucate with longitudinal crest; (**d**), tuberculate; (**e**), vermiculate-papillate; (**f**), rugulate; (**g**), longer spinose and globular elements; (**h**), shorter spinose and globular elements; (**i**), sheath-like; and (**j**), cable-like filaments. The drawings are based on Hennipman 1990; Large and Braggins 1991; Shalimov et al. 2013; Tryon and Lugardon 1991; van Uffelen 1993; and our own observations

**Fig. 2:**
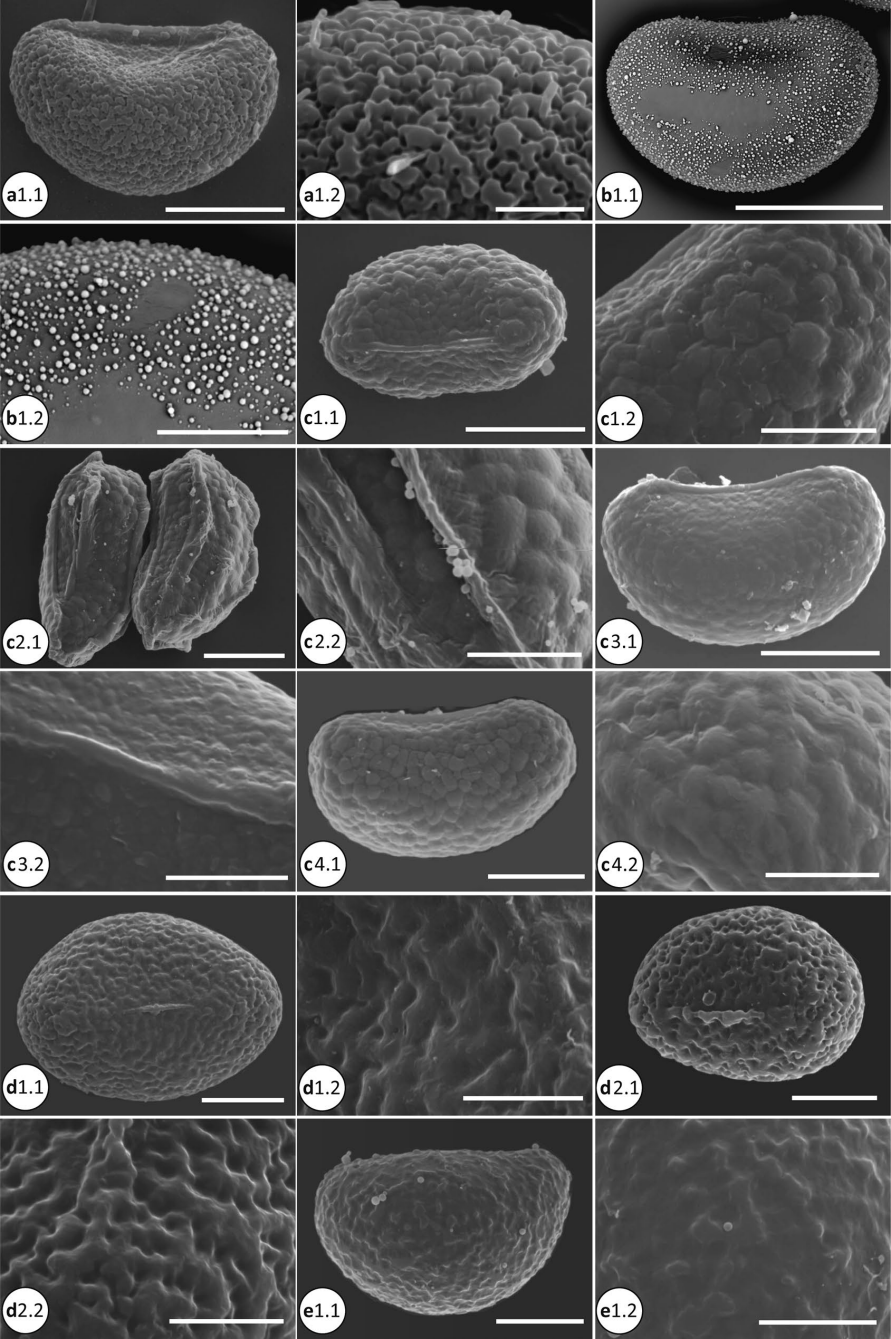

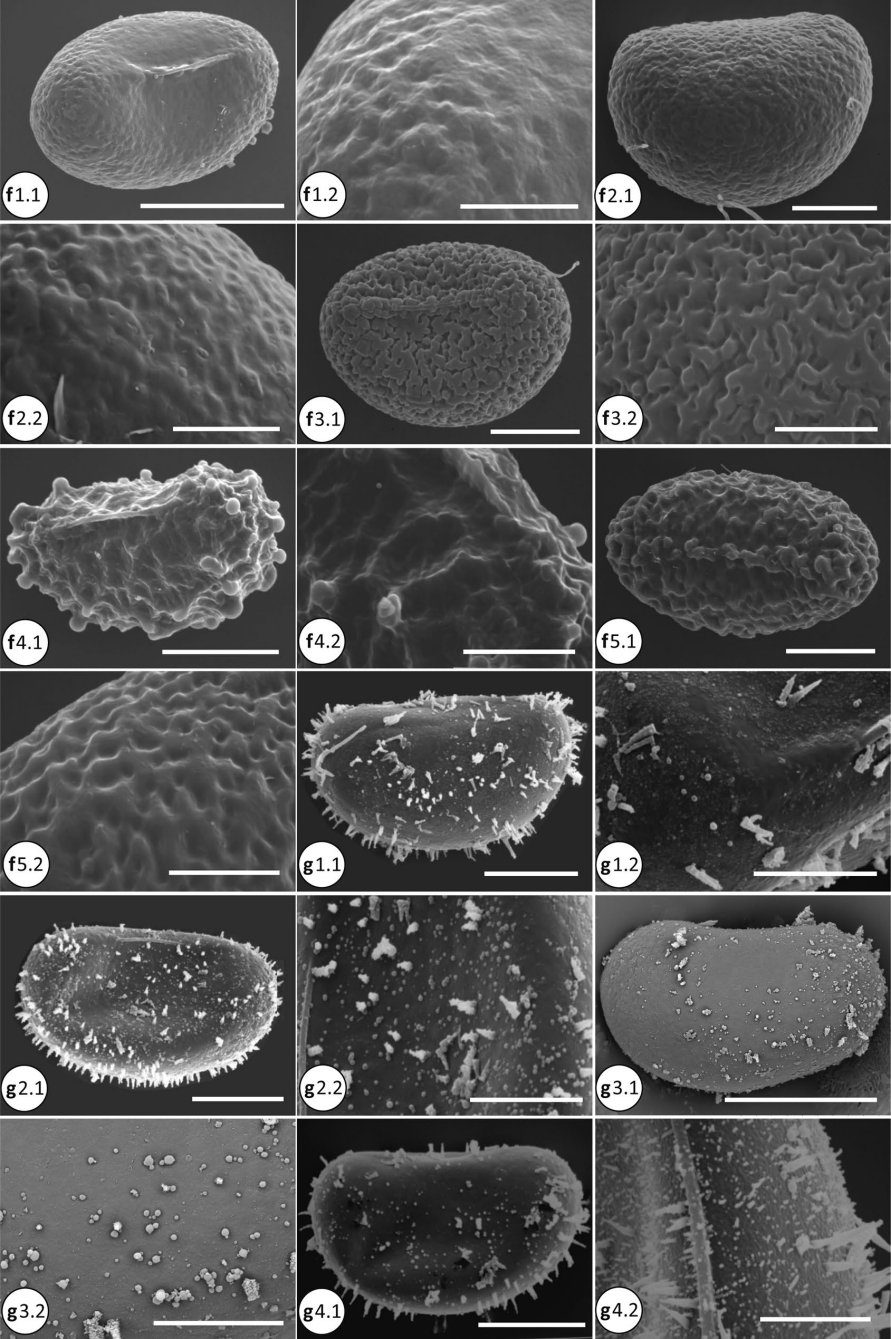

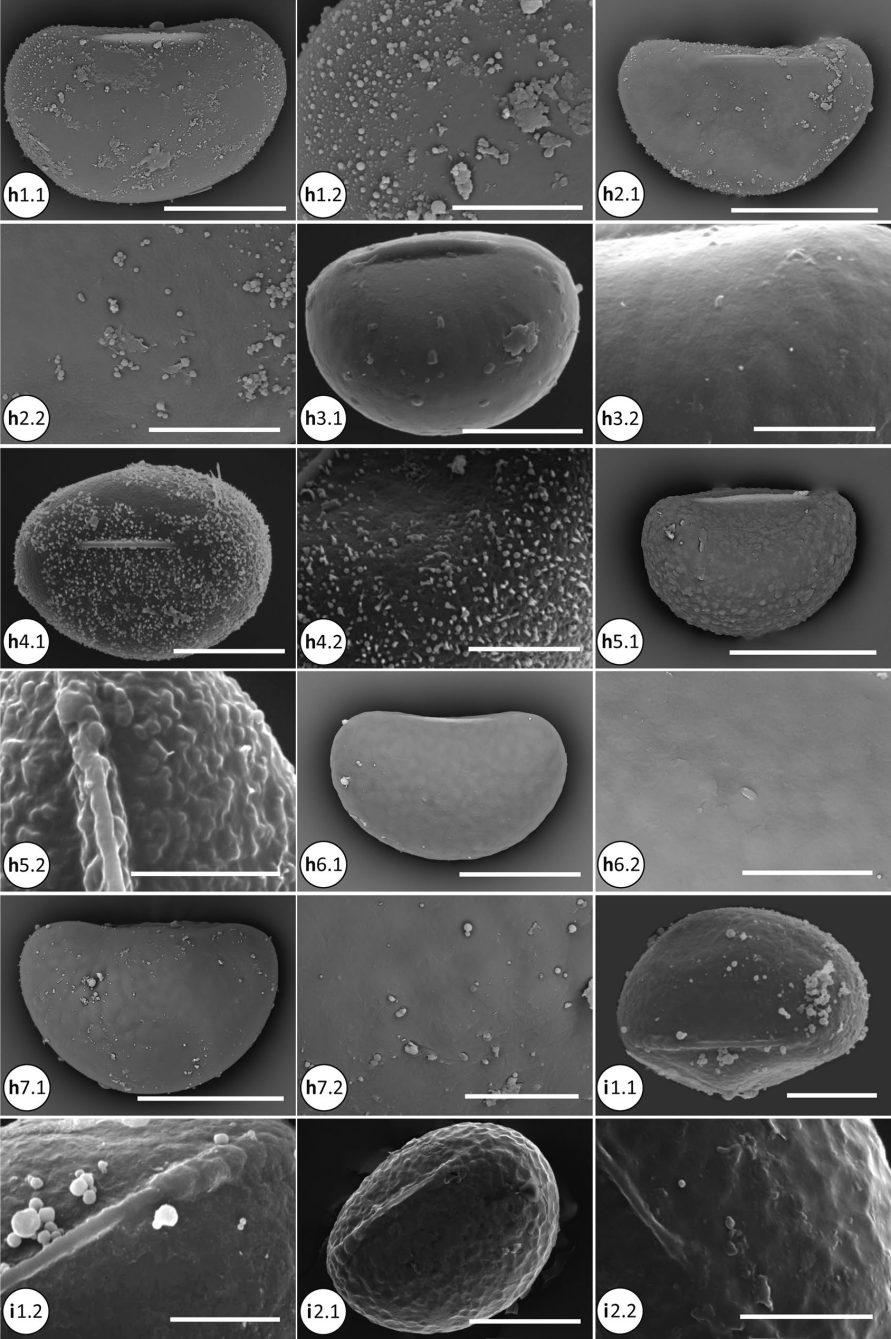

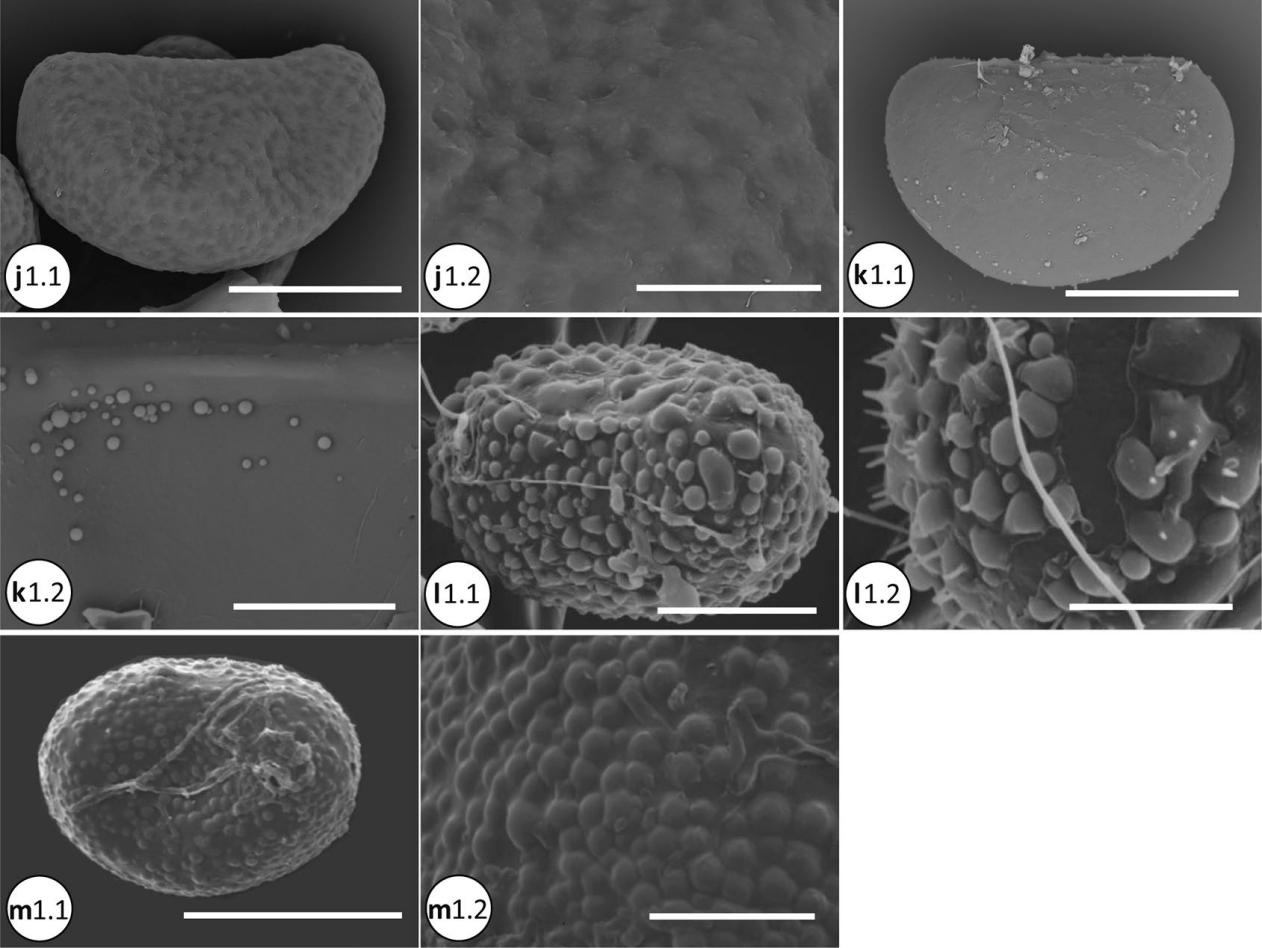
**a** Spore SEM of the microsoroid ferns. a1.1–a1.2, *Bosmania membranacea* (CC.Chen 249); a1.1, vermiculate-papillate ornamentation; a1.2. Detail of surface, the baculate is visible. b1.1–b1.2, *Dendroconche linguiforme* (SITW02028). c1.1–c1.2, *Goniophlebium amoenum* (CC.Chen 096). c2.1–c2.2, *G. argutum* (CC.Chen 105). c3.1–c3.2, *G. formosanum* (CC.Chen 016). c4.1–c4.2, *G. niponicum* var. *niponicum* (CC.Chen 159); verrucate surface with the inconspicuous membranous. d1.1–d1.2, *Lemmaphyllum microphyllum* (CC.Chen 002). d2.1–d2.2, *L. rostratum* (CC.Chen 014). e1.1–e1.2, *Lepidomicrosorium superficiale* (CC.Chen 008). Scale bars, 30 μm: b1.1; 20 μm: a1.1, c1.1, c2.1, c3.1, c4.1, d1.1, d2.1, e1.1; 10 μm: b1.2, c1.2, c2.2, c3.2, c4.2, d1.2, d2.2, e1.2; 5 μm: a1.2. **b** Spore SEM of the microsoroid ferns (continue). f1.1–f1.2, *Lepisorus clathratus* (CC.Chen 099). f2.1–f2.2, *L. miyoshianus* (TY.Tzi 720). f3.1–f3.2, *L. obscurevenulosus* (CC.Chen 350). f4.1–f4.2, *L. pseudoussuriensis* (CC.Chen 054). f5.1–f5.2, *L. thunbergianus* (CC.Chen 003). g1.1–g1.2, *Leptochilus decurrens* (Y.C.Liou 0047). g2.1–g2.2, *L. hemionitideus* (Y.C.Liou 2521). g3.1–g3.2, *L. pteropus* (P.F.Lu 29,763). g4.1-g4.2, *L. wrightii* (g4.1: CC.Chen 118; g4.2: CC.Chen 190). Scale bars, 50 μm: f1.1; 30 μm: g3.1; 20 μm: f2.1, f3.1, f4.1, f5.1, g1.1, g2.1, g4.1; 10 μm: f1.2, f2.2, f3.2, f4.2, f5.2, g1.2, g2.2, g3.2, g4.2. **c** Spore SEM of the microsoroid ferns (continue).h1.1–h1.2, *Microsorum cuspidatum* (FN287). h2.1–h2.2, *M. insigne* (P.F.Lu 27,122). h3.1–h3.2, *M. punctatum* (CC.Chen 149). h4.1–h4.2, *M. rubidum* (CC.Chen 113). h5.1–h5.2, *M. scolopendria* (h5.1: SITW02007; h5.2: CC.Chen 103). h6.1–h6.2, *M. steerei* (H.L.Chiang 2963). h7.1–h7.2, *M. thailandicum* (Y.L.Chang K013591, K013594). i1.1–i1.2, *Neolepisorus ensatus* (Y.N.Co 0393). i2.1–i2.2, *N. fortunei* (CC.Chen 049). Scale bars, 50 μm: h7.1; 30 μm: h1.1, h2.1, h5.1, h6.1; 20 μm: h3.1, h4.1, i1.1, i2.1; 10 μm: h1.2, h2.2, h3.2, h4.2, h5.2, h6.2, h7.2, i1.2, i2.2. **d** Spore SEM of the microsoroid ferns (continue). j1.1–j1.2, *Tricholepidium normale* (FN268). k1.1–k1.2, *Zealandia powellii* (SITW04893). l1.1–l1.2 (outgroup), *Aglaomorpha meyeniana* (CC.Chen 222). m1.1–m1.2 (outgroup), *Pyrrosia polydactyla* (CC.Chen 047). Scale bars, 50 μm: m1.1; 30 μm: j1.1, k1.1; 20 μm: l1.1; 10 μm: j1.2, k1.2, l1.2, m1.2

**Fig. 3:**
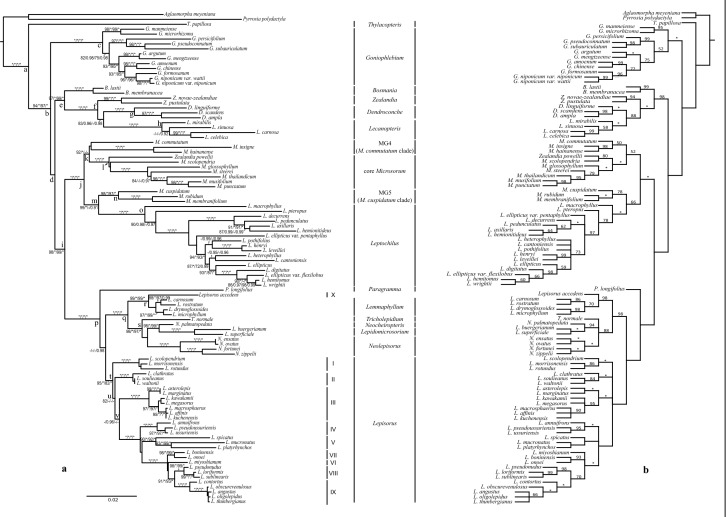
Phylogenetic relationships of the microsoroid ferns. (**a**) The optimal tree obtained in the ML analysis. Branch lengths correspond to the estimated number of substitution events. The first three values on the branches indicate the value of Shimodaira-Hasegawa-like approximate likelihood ratio test (SH-aLRT, %), *p* values of the Bayesian-like transformation of aLRT statistics (abayes), and ultrafast bootstrap analysis (UFBoot, %), respectively. The last value indicates posterior confidence values of BI (pp) generated with the MrBayes analyses using a combined matrix. (**b**) Tree obtained in the parsimony analysis, the numbers beside branches indicate bootstrap values. The asterisk, *, indicates branches with maximum values (1.00 or 100%) of the indices used for both trees; a dash, –, indicates low values: < 0.90 (90%) in abayes, and UFBoot, pp, and < 80% in SH-aLRT for tree (**a**). The nodes a–v on the tree (**a**) are used for Bayesian reconstruction with BayesTraits

**Fig. 4:**
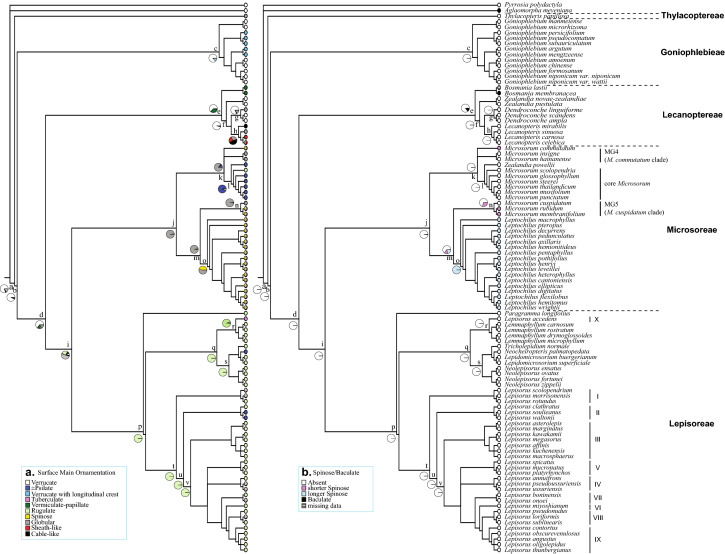
Optimization of spore characters based on Bayesian reconstruction using BayesTraits on the tree topology obtained in analysis with the program MrBayes. **a** surface main ornamentation, and (**b**) spinose or baculate projections. The detailed data for the pie charts is shown in Table S2. Groups I–X indicate subclades of *Lepisorus*

Some species may show variation between the samples and this also influenced our use of the terms. For example, terms retate and rugate indicated muri with or without anastomosing respectively (Lellinger 2002), but these ornamentation types can be observed in the different specimens of the same species, especially the species within the tribe Lepisoreae. To minimize these effects, the ornamentation was classified using general macro-characteristics. Surface ornamentation was scored (as illustrated in Fig. 1): (0) verrucate: width of surface projections greater than height (Punt et al. 2007) (Fig. 1a); (1) psilate or almost psilate: with a smooth surface (Lellinger 2002; Punt et al. 2007) (Fig. 1b); (2) verrucate with longitudinal crest (Shalimov et al. 2013) (Fig. 1c); (3) tuberculate: width of surface projections greater than or equal to height (Punt et al. 2007) (Fig. 1d); (4) vermiculate-papillate: mixture of winding projections and small protuberances (Lellinger 2002; Punt et al. 2007) (Fig. 1e); (5) rugulate: ornamentation in an irregular pattern that is intermediate between striate and reticulate (Punt et al. 2007) (Fig. 1f); (6) spinose: long and tapering pointed elements (Punt et al. 2007) (Fig. 1g-h); (7) globular: composed of globules as used in Tryon and Lugardon (1991) (Fig. 1g-h); (8) sheath-like: as sheaths used in Tryon and Lugardon (1991) (Fig. 1i); and (9) cable-like filamentous (Tryon and Lugardon 1991) (Fig. 1j).

In addition, the type of projections was scored as: (0) not spinose or baculate; (1) shortly spinose, with spine height ca. 1–2 X the width; (2) spinose, with spines distinctly higher than their width; (3) baculate. The data matrix had been summarized in Table S1.

### Phylogenetic analyses

The molecular dataset was analyzed using Maximum Likelihood (ML), parsimony as optimality criteria, and with Bayesian Inference (BI). For ML analysis, IQ-TREE 1.6.11 (Nguyen et al. 2015) was used as implemented on the W-IQ-TREE web server (http://iqtree.cibiv.univie.ac.at/; Trifinopoulos et al. 2016). We used the optimal partitioning scheme for phylogenetic analysis estimated by PartitionFinder v2.1.1 (Lanfear et al. 2017) at CIPRES Science Gateway (Miller et al. 2010), with the best fitting model selected using ModelFinder (Kalyaanamoorthy et al. 2017) as implemented in IQ-Tree. We evaluated the node support by 1000 ultrafast bootstrap replicates (UFBoot; Minh et al. 2013), Shimodaira-Hasegawa-like approximate likelihood ratio test (SH-aLRT; Guindon et al. 2010), and the Bayesian-like transformation of aLRT (aBayes; Anisimova et al. 2011) (Fig. 2).

BI analysis was implemented using MrBayes 3.2.7a (Ronquist et al. 2012) on the CIPRES, with the partitioned regions used. Markov chain Monte Carlo was run independently twice with one cold and three hot chains. In each run, chains were sampled every 1000 cycles. A total of 10 million generations were run and a majority rule consensus tree was calculated based on all trees sampled, excluding the first 25% of the sampled trees, which were discarded within the burn-in phase. This was examined using Tracer v. 1.6 (Rambaut and Drummond 2007) to ensure convergence of chains and sufficient sampling of generations. The posterior probabilities (PP) were calculated and presented using the majority rule consensus tree.

The parsimony analyses were conducted using the heuristic search algorithms of NONA 2.0 (Goloboff 1998) with the WinClada (Nixon 2002) shell under the following settings: maximum trees kept (hold) = 100,000; number of replications (mult*N) = 1000; starting trees per rep (hold/) = 100; random seed = time; search strategy = multiple TBR + TBR (mult*max); unconstrained search. The obtained trees were examined and analysed under different optimizations using WinClada. Bootstrap value was calculated using 1000 replications and 10 search replications with one starting tree per replication and without tree bisection- reconnection (TBR). All character states were treated as unordered and equally weighted, and gaps were treated as missing data.

### Ancestral state reconstruction of spore characters

We calculated probabilities of ancestral states in BayesTraits version 3.0 (Pagel and Meade 2006), and mapped on the consensus tree obtained from MrBayes. To incorporate phylogenetic uncertainty, we used R to choose, at random, 100 post burn-in trees from the MrBayes analysis, with the information of branch-length included. Ancestral states were reconstructed for 22 nodes (a-v in Figs. 3a, 4) for each character. We used the “Multistate” model. A reversible-jump hyperprior with an exponential prior was used to reduce uncertainty of choosing priors in the MCMC analysis. The option “AddNode” was used to find the proportion of the likelihood associated with each of the possible states at each node. The MCMC run was performed with 10 million iterations. Chains were sampled every 1000 iterations with a burn-in of 5 million iterations.

## Results

### Description of spore ornamentation

Spores of the microsoroid ferns were monolete with bilateral symmetry. The shape was elliptic-oblong in polar view, and plano- to concavo- convex in equatorial view. Totally ten types of the main surface characters and three types of projections were described here.

1. Tribe Goniophlebieae C.C. Chen and H. Schneider
2. 1a. *Goniophlebium* (Blume) C. Presl (Fig. 2 c1–c4)
  Twelve species were included: *Goniophlebium amoenum* (Wall. ex Mett.) Bedd., *G. argutum* (Wall. ex Hook.) J. Sm. ex Hook., *G. chinense* (Christ) X.C. Zhang, *G. formosanum* (Baker) Rodl-Linder, *G. manmeiense* (Christ) Rodl-Linder, *G. mengtzeense* (Christ) Rodl-Linder, *G. microrhizoma* (C.B. Clarke ex Baker) Bedd., G. *niponicum* (Mett.) Bedd. var. *niponicum*, *G. niponicum* var. *wattii* (Bedd.) Bedd., *G. persicifolium* (Desv.) Bedd., *G. pseudoconnatum* (Copel.) Copel., and *G. subauriculatum* (Blume) C.Presl. The range of spore size was 17–52 × 34–82 μm. Surface ornamentation was verrucate, with or without the longitudinal crest. The former (verrucate with longitudinal crest) can be observed in two subclades containing *G. argutum* and *G. persicifolium*, respectively (Fig. 4a; e.g. Figure 2, c2), with spores of the other species without such distinctive structures (e.g. Figure 2 c1, c3–c4).
3. Tribe Lecanoptereae C.C. Chen and H. Schneider
4. 2a. *Bosmania* Testo (Fig. 2 a1)
  Two species were included: *Bosmania lastii* (Baker) Testo and *B. membranacea* (D. Don) Testo. The range of spore size was 20–54 × 37–70 μm. Surface ornamentation of both species was vermiculate-papillate. In addition, we observed bacula on the spore surface of *B. membranacea* (D. Don) Testo (Fig. 2 a).
5. 2b. *Dendroconche* Copel. (Fig. 2 b1)
  Three species were included: *Dendroconche ampla* (F. Muell. ex Benth.) Testo, Sundue, and A.R. Field, *D. linguiforme* (Mett.) Testo, Sundue, and A.R. Field, and *D. scandens* (G. Forst.) Testo, Sundue, and A.R. Field. Spores of *D. linguiforme* were larger (30–60 × 45–105 μm) than those of the other two species. The surface ornamentation of *D. linguiforme* showed many small globular elements on the psilate surface (Fig. 2 b1; plate 2:a in Bosman 1991), while the other two species had verrucate ornamentation (Figs. 116.10 in Tryon and Lugardon, 1991; Fig. 1:D in Large et al. 1992).
6. 2c. *Lecanopteris* Reinw. ex Blume
  Four species were included: *Lecanopteris carnosa* (Reinw.) Blume, *L. celebica* Hennipman, *L. mirabilis* (C. Chr.) Copel., and *L. sinuosa* (Hook.) Copel. The range of the spore size was 32–38 × 42–60 μm. The spores of this genus usually have a psilate surface with various ornamentations including (a) cable-like filaments (Figs. 118.17–18 in Tryon and Lugardon 1991; Fig. 2.7:g in Hennipman 1990), and (b) sheath-like structures (Figs. 118.7–11 in Tryon and Lugardon 1991). The former can be seen only in *L. mirabilis*, rather unique among microsoroid ferns.
7. 2d. *Zealandia* Testo and A. R. Field (Fig. 2 k1)
  Three species were included, *Zealandia novae-zealandiae* (Baker) Testo and A. R. Field, *Z. pustulata* (G. Forst.) Testo and A. R. Field, and *Z. powellii* (Baker) Testo and A. R. Field. The range of spore size was 14–44 × 31–70 μm. Surface ornamentation of the former two species was mainly verrucate (Fig. 1:A and C in Large et al. 1992), while the ornamentation of *Z. powellii* was psilate with some globular elements (Fig. 2k1).
8. Tribe Lepisoreae Ching ex E Hennipman, P Veldhoen and KU Kramer
  Spore ornamentation was quite uniform in all species of this tribe. They mainly showed rugulate ornamentation, with some subtle variation between genera, subclades, and species. Among the seven genera, particularly *Lepisorus* showed some diversity in the rugulate ornamentation.
9. 3a. *Lemmaphyllum* C. Presl (Fig. 2 d1–d2)
  Four species were included: *Lemmaphyllum carnosum* (Wall. ex J. Sm.) C. Presl, *L. drymoglossoides* (Baker) Ching, *L. microphyllum* C. Presl, and *L. rostratum* (Bedd.) Tagawa. The range of spore size was 25–77.5 × 39–102.5 μm. The surface ornamentation of these species was deep rugulate, sometimes mixed with tuberculate ornamentation (Fig. 2.3:d in Hennipman 1990).
10. 3b. *Lepidomicrosorium* Ching and K.H.Shing (Fig. 2e1)
  Two species were included: *Lepidomicrosorium buergerianum* (Miq.) Ching and K.H. Shing and *L. superficiale* (Blume) L. Wang. The range of spore size was 23–60 × 34–75 μm, with rugulate as the main ornamentation type.
11. 3c. *Lepisorus* (J.Sm.) Ching (Fig. 2 f1–f5)
  Thirty-one species were included, and the range of spore size was 20–72.5 × 32–107.5 μm. Three types of ornamentation included psilate, tuberculate, and as the most common, rugulate spores found in this genus (Fig. 4). Some variation of the rugulate character can be observed including mixing with tuberculate spread all over the surface, such as in *L. morrisonensis* H. Itô and *L. scolopendrium* Mehra and Bir (Fig. 2 b, c in Kholia et al. 2012) species of the subclade I; rugulate with fused parts especially on the opposite side of the laesurae, as in *L. pseudoussuriensis* Tagawa (Fig. 2, f4) of the Group IV. Some species had a foveolate-rugulate ornamentation, such as *L. clathratus* Ching (Fig. 2 f1; Plate E:6 in Mitui 1977) within Group II, as well as several species of the Group III.
12. 3d. *Neocheiropteris* H. Christ
  One species, *Neocheiropteris palmatopedata* (Baker) Christ, was included. The spore size was 29–38 × 44–54 μm. Surface ornamentation of *N. palmatopedata* (Baker) Christ was psilate, with some globular elements on the surface (Figs. 121.3 in Tryon and Lugardon 1991).
13. 3e. *Neolepisorus* Ching (Fig. 2 i1–i2)
  Four species were studied: *Neolepisorus ensatus* (Thunb.) Ching, *N. fortunei* (T.Moore) Li Wang, *N. ovatus* (Wall. ex Bedd.) Ching, and *N. zippelii* (Blume) L. Wang. The range of spore size was 20–52.5 × 28–82.5 μm, and *N. ensatus* had larger spores than the other three species. The ornamentation was rugulate, with globular elements also found on the spore surface of *N. ensatus* (Fig. 2 i1).
14. 3f. *Paragramma* (Blume) T. Moore
  One species, *Paragramma longifolia* (Blume) T. Moore, was studied. The spore size was 35–41 × 50–66 μm. The ornamentation of this species was rugulate mixed with few tuberculate ornamentation (Fig. 2.3:f in Hennipman 1990; Fig. 114.1 in Tryon and Lugardon 1991).
15. 3 g. *Tricholepidium* Ching (Fig. 2 j1)
  One species, *Tricholepidium normale*, was included, with a spore size of 32–45 × 38–67 μm. The surface ornamentation was rugulate (Fig. 2j1; Figs. 120.6 in Tryon and Lugardon 1991).
16. Tribe Microsoreae V.N.Tu
17. 4a. *Leptochilus* Kaulf. (Fig. 2 g1–g4)
  Seventeen species were included. The range of spore size was 17.5–47.5 × 32.5–81 μm. The surface ornamentation of this genus mainly included different spinose quantities mixed with globular elements, and the height of spinose was greater than their width. The spinose proportion to globular elements exposed the differences between species. Most species had mainly spines; but some, such as *L. pteropus* (Blume) Fraser-Jenk., can be described as predominantly globular (Fig. 2 g3). In addition, we observed some species to have granulate material spread over the surface including spines and globular elements, making the surface coarse (e.g., Fig. 2 g1.2, g2.2; Figs. 119.2 in Tryon and Lugardon 1991).
18. 4b. *Microsorum* Link (Fig. 2 h3, h5–h7)
  Six species of the *Microsorum* sensu stricto were included: *M. musifolium* (Blume) Copel., *M. punctatum* (L.) Copel., *M. scolopendria* (Burm. f.) Copel., *M. steerei* (Harr.) Ching, *M. thailandicum* T. Booknerd and Noot., and *M. glossophyllum* Copel. The range of spore size was 20–61 × 34–86 μm. The main surface ornamentation of this genus was psilate, except for *M. scolopendria*. The ornamentation of *M. scolopendria* showed variation, with the specimens from Cameroon, Sumatra, and New Guinea having slightly rugulate spore surface (Figs. 122.1–4 in Tryon and Lugardon 1991); whereas specimens from Japan and Taiwan had rugulate-tuberculate spores (Plate B:5, Plate D:8 in Mitui 1977; Fig. 2 h5).
19. 4c. MG4, *Microsorum commutatum* clade (Fig. 2 h2)
  Three species were included in this clade: *M. commutatum* (Bl.) Copel., *M. insigne* (Blume) Copel., and *M. hainanense* Noot. The range of spore size was 20–61.5 × 34–94.5 μm. The spores of *M. hainanense* were larger compared to the other species in this clade. The surface ornamentation of *M. commutatum* had both globular elements and spines of comparable size (shorter spinose) (Plate IV:8 in Van Uffelen 1993); both *M. insigne* and *M. hainanense* had mainly globular elements on the psilate surface (Fig. 2 h2; Plate CVII:9–10 in Wang 2001; Plate I:8–9 in Shi 2002).
20. 4d. MG5, *Microsorum cuspidatum* clade (Fig. 2h1, h4)
  This clade includes three species: *Microsorum cuspidatum* (D. Don) Tagawa, *M. rubidum* (Kunze) Copel., and *M. membranifolium* (R. Br.) Ching. The range of spore size was 20–57 × 35–105 μm. The main surface ornamentation types were globular, both with and without shorter spines. Spore surface of *M. cuspidatum* had only globular elements (Fig. 2 h1), whereas *M. rubidum* and *M. membranifolium* had both short spinose and globular elements (Fig. 2 h4; Figs. 122.8 in Tryon and Lugardon 1991). In addition, also foveolate surface was observed in *M. rubidum* (Fig. 2 h4).
21. Tribe Thylacoptereae C.C. Chen and H. Schneider
22. 5a. *Thylacopteris* Kunze ex J. Sm.
  One species, *Thylacopteris papillosa* (Blume) J.Sm., was included with a spore size of 42 × 54–66 μm. The ornamentation was psilate with many globular elements attached (Fig. 3 c-d in Rödl-Linder 1994).
  We included two outgroup species, *Aglaomorpha meyeniana* Schott and *Pyrrosia polydactyla* (Hance) Ching. Both species had verrucate surface ornamentation (Fig. 2l1, m1).

### Phylogenetic analyses

In general, the consensus trees obtained from the ML analyses (Fig. 3a) and BI analyses (Fig. 4) were congruent except the MG4 (*Microsorum commutatum* clade), IV-V subclades of *Lepisorus*, and *Tricholepidium normale* (D. Don) Ching. The former two are part of the polytomy in BI topology (Fig. 4), the latter, *T. normale* located in the basal position of *Neocheiropteris-Lepidomicrosorium- Neolepisorus* in ML topology (Fig. 3a), but in the basal position of *Neocheiropteris-Lepidomicrosorium* in BI topology (Fig. 4). In order to simplify presentation of the results, the values of posterior probabilities of the BI analyses were illustrated on the ML topology (Fig. 3a). In the parsimony analyses, the molecular dataset had 5814 characters, with 1459 of those being parsimony-informative. Thirty equally parsimonious trees of length 5535 (CI = 50, RI = 72) were obtained. The strict consensus tree included several polytomies: subclades within *Leptochilus*, clades *Tricholepidium*—*Neolepisorus*, and subclades of *Lepisorus* (Fig. 3b).

### Spore character evolution

The number of globular elements on the spore surface varied to great extent between species, only the species with high density globular (usually more than 150) were scored as globular state in Fig. 4a. In Bayesian analyses, most nodes showed significant posterior probability values in at least one character state (Table S2). The ancestral state for the spore surface ornamentation was verrucate for the microsoroid ferns, present in the basal nodes a–g, including Goniophlebieae and Lecanoptereae (PP = 0.8649 and PP = 0.6348, Table S2). For Microsoreae, psilate and globular ornamentations were reconstructed as the ancestral states, the former was specific for the node l (i.e. core *Microsorum*), and the latter at nodes j, k, m, n, o, corresponding to Microsoreae*,* MG4 plus core *Microsorum*, MG5 plus *Leptochilus*, MG5, and *Leptochilus*, respectively (Fig. 4a). For tribe Lepisoreae (nodes p–v), rugulate was the ancestral state at all studied nodes (Table S2, Fig. 4). Of all the microsoroid ferns clades, species of Lecanoptereae showed most variation in their spore ornamentation, with five types represented: vermiculate-papillate, verrucate, globular, sheath-like, and cable-like filaments (Fig. 4a).

For type of projections, the lack of spinose/baculate surface was the most common ancestral state at all nodes except for node o (i.e. *Leptochilus*), at which the longer spinose reconstructed as a synapomorphy (PP = 0.9996, Table S2; Fig. 4b).

## Discussion

### Morphology and evolution of spore ornamentation

Our observations are mostly congruent with earlier reports about spore surface ornamentation of the microsoroids. Ten different types of spore surface ornamentations formed by spore walls were observed in this study (Fig. 1; Fig. 4a). Some of the ornamentations are formed by exospore, such as verrucate of Goniophlebieae and Lecanoptereae (Large and Braggins 1991; Large et al. 1992; Tryon and Lugardon 1991); some ornamentations by perispore, such as sheath-like and cable-like filaments of *Lecanopteris*, and spinose of Microsoreae (Hennipman 1990; Tryon and Lugardon 1991; van Uffelen 1993,1997); with some determined by both exospore and perispore, such as verrucate with longitudinal crest of Goniophlebieae (Tryon and Lugardon 1991). This demonstrates the diversity and complexity of the microsoroid ferns, which is consistent with the classification regarding sporoderm by Tryon and Lugardon (1991). However, as already mentioned above, more complete comparison of exospore and perispore requires additional data using TEM, since TEM sections may provide more precise estimates than sections obtained via breaking of the spore wall during the preparation for the SEM. There have been numerous efforts to understand the spore wall structure of Polypodiaceae (e.g., Hennipman 1990; Tryon and Lugardon 1991; van Uffelen 1993), but the TEM observations of microsoroids are still insufficient. In order to understand and compare different species of the group also ontogeny of the spores should be studied in detail. This is why, also in our analyses, we treated the visible surface ornamentation as one character.

Reconstruction of the ancestral state shows that verrucate is most likely the ancestral state of the spore surface ornamentation of the microsoroid ferns, exhibited in the basal nodes (a–g), including tribes Goniophlebieae and Lecanoptereae (Table S2, Fig. 4a). All studied species of Goniophlebieae (*Goniophlebium*) have verrucate surface, with or without longitudinal crests, and present in different subclades (Fig. 4a). Clades *Zealandia* and *Dendroconche* of Lecanoptereae also have verrucate ornamentation, however, the shape and size of verrucae differ from those found in *Goniophlebium*. Verrucae of *Zealandia* are more irregular, while in *Dendroconche ampla* and *D. scandens*, they are relatively small micro-verrucae (Large et al. 1992; Tryon and Lugardon 1991). For the other two genera of Lecanoptereae, *Lecanopteris* exhibits cable-like filaments as the ancestral state, but with only low support value (PP = 0.4105, Table S2); *Bosmania* has vermiculate-papillate as the main ornamentation (Fig. 4a), which has been considered a special exospore type in the previous studies (Hennipman 1990; van Uffelen 1997). Among the studied genera/clades of the microsoroid ferns, spore ornamentation of *Lecanopteris* is relatively diverse and unique, including cable-like filaments, sheath-like, and globular elements (Fig. 4a). The former two ornamentation types are unique types found only in this genus (Tryon and Lugardon 1991), and likely autapomorphies in the microsoroid ferns (Fig. 4a). It is reasonable to suppose that spore diversity of *Lecanopteris* may be related to their relationship with ants, since some studies show that the spore of *Lecanopteris* may be transported and utilized by them (Tryon 1985; Tryon and Lugardon 1991).

Globular ornamentation is reconstructed as the ancestral state for tribe Microsoreae, except for core *Microsorum*, where psilate is the main ornamentation type (PP = 0.9316, Table S2). Unlike the relatively simple surface of core *Microsorum*, the other three genera/clades (MG4, MG5, and *Leptochilus*) exhibit numerous globular elements, with or without spinose on the surface, that might represent a synapomorphy (Fig. 4). For *Leptochilus*, not only globular but spinose are likely ancestral states, with posterior probabilities of 0.5215 and 0.4730, respectively (Table S2). Spinose projections of *Leptochilus* are usually larger and less uniform, which may be a synapomorphy. Spores in the clades of MG4 and MG5 also have spinose surfaces, but not in all species. Spinose projections of these two clades are smaller differing from species of *Leptochilus* (Fig. 4b).

There are three spore surface ornamentation types observed in tribe Lepisoreae: rugulate, tuberculate, and psilate. Tuberculate ornamentation typically mixes with rugulate, except in *Lepisorus accedens* (Fig. 4a), with only a few species have tuberculate and psilate ornamentations. Rugulate is reconstructed as the ancestral state for seven studied nodes (PP > 0.93, Table S2), and may represent a synapomorphy of tribe Lepisoreae (Fig. 4a).

### Taxonomic considerations

Spore surface types of the microsoroid ferns are generally congruent with the phylogenetic relationships obtained using plastid DNA sequence data. There are five tribes currently accepted within the microsoroid ferns (Chen et al. 2020). Tribe Thylacoptereae has only one species and it shows globular ornamentation, tribe Lecanoptereae shows the most diversity in spore surface ornamentation with six types. Of the other three tribes, Microsoreae has four, Lepisoreae three, and Goniophlebieae two types, respectively (Fig. 4a).

Lecanoptereae contains four genera: *Bosmania*, *Lecanopteris*, *Dendroconche*, and *Zealandia*. The vermiculate-papillate ornamentation of *Bosmania* is unique and can be distinguished from other Polypodiaceae (Hennipman 1990; Van Uffelen 1997). Spores of *Lecanopteris* show diversity, especially the cable-like filaments of *L. mirabilis* are distinct, and have not been reported in other species (Hennipman 1990; Tryon and Lugardon 1991). The four species of *Lecanopteris* studied differ from each other in their spore ornamentation. It would be important to explore this unusually labile nature of the ornamentation more in detail, and how it relates to the possible functional adaptation of spores (Tryon and Lugardon 1991). Genera *Dendroconche* and *Zealandia* have species found mostly in Oceania, with verrucate as the main spore ornamentation, except for *D. linguiforme* and *Z. powellii*. The former has globular spore surface, while the latter has psilate ornamentation (Fig. 4a). The position of *Z. powellii* varies, as it has been proposed to belong to both Microsoreae and Lecanoptereae (Chen et al. 2020; Nitta et al. 2018; Testo et al. 2019). In our analyses *Z. powellii* (sample from Solomon Islands) belongs to core *Microsorum* of Microsoreae, and its psilate ornamentation is similar to most species of core *Microsorum* also highlighting close relationship (Fig. 2 k1; Fig. 4a). However, this difference of position may also be caused by misidentification. The sequence data show differences between the specimens from Solomon Islands and Moorea respectively (Chen et al. 2020; Nitta et al. 2018). Further study is needed for reliable identification of these specimens and the type. In the same way, different ornamentations observed for the spores of *M. scolopendria* may be due to misidentification, specimens confused with *M. grossum*. Both species are morphologically similar and have overlapping ranges, with the former species can occur further north (Possley and Howell 2015).

In addition to core *Microsorum*, Microsoreae also includes *Leptochilus*, MG4 and MG5 clades (Chen et al. 2020). Of these four genera/clades, species of *Leptochilus* consistently have long spinose and globular elements as the main surface ornamentation (Fig. 1 g) of their spores, but the number of the spinose and globular elements differs between species. For example, *L. pteropus* and *L. macrophyllus* have more globular than spinose elements (Fig. 2 g3) (Tryon and Lugardon 1991, Figs. 116.3–4). *Leptochilus pteropus* has previously been placed in various genera (*Microsorum*, *Kaulinia*, and *Colysis*) based on the macromorphology (e.g., Bosman 1991; Fraser-Jenkins 2008; Nayar 1964; Nooteboom 1997). *Leptochilus* has recently been confirmed as the genus where this species belongs on the basis of molecular data (Zhang et al. 2019), and our spore data are consistent with this placement. Unlike *Leptochilus*, the spore ornamentation of core *Microsorum* is mainly psilate with a few globular, and without spinose elements. The phylogenetic position of both MG4 and MG5 clades has been studied recently (Chen et al. 2020). Our results show a similar topology except for the location of *M. hainanense*, which is in MG4 clade in our study with weak support value. Unfortunately, spore data cannot differentiate the two clades. Species within both MG4 and MG5 clades have globular elements as surface ornamentation, with or without spinose elements. When spines are present they are smaller than those seen in Leptochilus (Figs. 1 h, 4b). Based on the spore data these two clades differ from *Microsorum* and *Leptochilus*.

Lepisoreae contains seven genera: *Lemmaphyllum*, *Lepidomicrosorium*, *Lepisorus*, *Neocheiropteris*, *Neolepisorus*, *Paragramma*, and *Tricholepidium* (Chen et al. 2020), with *Lepisorus* divided into ten subclades (Fig. 3a). The spore ornamentation is mainly rugulate and seems to be quite consistent in this tribe, with only a few species showing the other two types (Fig. 4a). For example, *L. accedens* has tuberculate spore surface and is located in the *Lemmaphyllum*, according to our study (Fig. 3), however, with only weak support value based on molecular data (aLRT = 4.5%/aBayes = 0.57/UFBoot = 57.0%). The location of *L. accedens* differs from those found in previous studies (e.g., Chen et al. 2020; Zhao et al. 2019), this may be due to smaller sampled sizes in this study (Wei et al. 2017). The other species having tuberculate type are mixed with rugulate type, all of these can be found in the *Lepisorus* clade (Fig. 4a). Three species, *Neocheiropteris palmatopedata*, *Lepisorus soulieanus* (Christ) Ching and S.K. Wu and *L. waltonii* (Ching) S.L. Yu have a relatively smooth spore surface (Fig. 4a). The former is one of two species in the small genus *Neocheiropteris* (PPG I 2016), and the latter two species belong to clade II of *Lepisorus*. Another species of the clade II, *L. clathratus*, has slightly rugulate exospore (Fig. 2), and has been described also as psilate/smooth in some studies (Devi 1981; Kholia et al. 2012). Rugulate spore surface ornamentation is common in *Lepisorus* with different rugulate levels between subclades or species, these spore types are not a synapomorphy for this genus. Among ten subclades, species of the subclade X (i.e., *L. accedens*) have only tuberculate ornamentation; another nine subclades include rugulate plus tuberculate type in clades I, IV, VII, and VIII (e.g., Figure 2f4), those with slightly rugulate or psilate type are found in subclades II, III, and VI (e.g., Fig. 2 f1–f2), and subclades V and IX have moderately rugulate surface (e.g., Fig. 2 f3, f5). Descriptions of the spore surface of the species of *Tricholepidium* vary between different studies. The spore ornamentation of *T. normale* from Yunnan, China is psilate (Tryon and Lugardon 1991, under name *Microsorum normale*), or granulate (Wang 2001, under name *T. angustifolium*), but material from India shows baculate structure (Nayar and Devi 1964, under name *Microsorum normale*). The specimen we studied is from India, showing a rugulate surface (Fig. 2 j1). The subclades of *Lepisorus* and the genera of Lepisoreae, cannot be clearly distinguished based on their spore ornamentation. Zhao et al. (2019) recently treated species of Lepisoreae as *Lepisorus* sensu lato, and our observations of the spore ornamentation are not in conflict with this.

Classification of Goniophlebieae has varied in the past. It has either been treated as one genus (Kreier et al. 2008; PPG I 2016), or has been divided into several smaller genera, including *Goniophlebium* sensu stricto, *Metapolypodium*, *Polypodiastrum*, and *Polypodiodes* (Zhang et al. 2013). The spores of all species of Goniophlebieae have verrucate ornamentation, with or without the membraneous crest. Verrucate with membraneous crest is found in two subclades, one subclade contains *G. persicifolium*, *G. pseudoconnatum*, and *G. subauriculatum*, while another subclade contains *G. argutum* and *G. mengtzeense* (Fig. 4a). The former subclade belongs to *Goniophlebium* sensu stricto in the classification using small segregate genera, while the latter subclade belongs to *Polypodiastrum*, respectively.

## Conclusions

Spore surface ornamentation has been shown to be informative and useful also for phylogenetic studies (Schneider et al. 2009). Here we explored spore ornamentation of the microsoroid ferns and its taxonomic value, and based on our analysis, the ancestral state of the microsoroids spores surface appears to be verrucate. This surface ornamentation can be found in the genera of Goniophlebieae and Lecanoptereae. For the tribes Microsoreae and Lepisoreae the ancestral states of the spore surface ornamentation seem to be with globular elements and rugulate, respectively. Spore surface ornamentation types generally seem to be congruent with the clades found in the phylogenetic analyses based on molecular data, and this character can be used to distinguish genera and tribes of the microsoroid ferns, or even species in some cases, such as *Lecanopteris mirabilis*. Tribe Lecanoptereae shows most diversity in spore surface ornamentation, with three of the five ornamentations, vermiculate-papillate, sheath-like, and cable-like filaments, unique in the microsoroid species. The latter two ornamentations types are found in particular in *Lecanopteris*. This diversity of spore ornamentations types might prove to be useful in studies exploring the possible functional adaptation of microsoroid spores.

## Electronic supplementary material

Below is the link to the electronic supplementary material.

Supplementary file1 (PDF 459 KB)
