# Supplementary material for: On the spore ornamentation of the microsoroid ferns (microsoroideae, polypodiaceae)
Source: J Plant Res. 2020 Nov 29;134(1):55–76. doi: 10.1007/s10265-020-01238-4 (PMC7817562; doi:10.1007/s10265-020-01238-4)
Supplement: Supplementary file 1 — Supplementary file1 (PDF 459 KB) [file 10265_2020_1238_MOESM1_ESM.pdf]

Electronic supplementary materials

Title:

On the Spore Ornamentation of the Microsoroid Ferns (Microsoroideae, Polypodiaceae)

Authors:

Chi-Chuan Chen, Ho-Yih Liu, Cheng-Wei Chen, Harald Schneider, Jaakko Hyvönen

Journal:

Journal of Plant Research

Corresponding author:

Jaakko Hyvönen

Finnish Museum of Natural History (Botany) & Organismal & Evolutionary Biology; PO Box 7, FI-00014 University of Helsinki, Finland

E-mail address: jaakko.hyvonen@helsinki.fi

Content:

Tables S1–S2

Table S1. List of material used for the spore surface character analyses given as taxon name, SEM/TEM specimen data including locality, collector or specimen number with herbarium where deposited, the data source (this study or related literatures), number of photos (this study or earlier publications); and scored spore data for this study, with the last column showing the spore size. The number of data scoring described in the Terminology section of text. The dash indicates missing data. Abbreviation: E, equatorial diameter; P, polar diameter; Spi, spinose; Surf, Surface ornamentation.

Taxon	SEM specimens	Data source	Micrographs	Surf.	Spi.	P×E (μm)
Bosmania lastii (Baker) Testo	-	Bosman (1991)	-	4	-	25–30 × 40–50
Bosmania membranacea (D. Don) Testo	Taiwan: Kaohsiung; CC.Chen 249 (SYSU)	This study	Fig.1 A1.1-A1.2	4	3	20–54 × 37–70
	Bhutan; Ludlow et al. 17052	Tryon & Lugardon (1991)	Figs. 120.12-13			
	Phusomsaeng BKF 46336	Bosman(1991)	Plate 2:c			
	Mainland China: Sichuan; W.P.Fang 6881 (PE)	Wang (2001)	Plate CVI 9-12			
	-	Hennipman (1990)	-			
Dendroconche ampla (F. Muell. ex Benth.) Testo, Sundue, & A.R. Field	Australia: Queensland; Smith 03371	Tryon & Lugardon (1991)	Figs. 116.10	0	0	36 × 52
Dendroconche linguiforme (Mett.) Testo, Sundue, & A.R. Field	Solomon: Obubulu Village; Y.H.Chang et al. SITW02028 (TAIF 443083)	This study	Fig.1 B1.1-B1.2	7	0	30–60 × 45–105
	Anonymus, HBL 6478	Bosman (1991)	Plate 2:a			
Dendroconche scandens (G. Forst.) Testo, Sundue, & A.R. Field	New Zealand: Albany, Tindale; M.F.Large (AKU 22122)	Large et al. (1992)	Fig. 1:D	0	0	21–41× 38–62
	New Zealand: Paparoa range; M.F.Large MFL157 (AKU 22121)	Large & Braggins (1991)	Fig. 227			
Goniophlebium amoenum (Wall. ex Mett.) Bedd.	Taiwan: Hualien; CC.Chen 096 (SYSU)	This study	Fig.1 C1.1-C1.2	0	0	17–52 × 34–82
	India: Sikkim; Hara et al.	Tryon & Lugardon (1991)	Figs. 131.5			
	Vietnam: Thai Nguyen	Shalimov(2013)	Fig. 1			
	Mainland China: Guizhou; Q.X.Wang et al.	Wang (2001)	Plate CII 3			
	Mainland China: Guizhou; Q.X.Wang et al. s. n. (GBG)	Jiang et al. (2010)	Plate I 6			
Goniophlebium argutum (Wall. ex Hook.) J. Sm. ex Hook.	Taiwan: Taichung; CC.Chen 105 (SYSU)	This study	Fig.1 C2.1-C2.2	2	0	23–45 × 40–65
	Mainland China: Guizhou; Tsiang 5911	Tryon & Lugardon (1991)	Figs. 132.4			
	Mainland China: Yunnan; No. 10964, Henry A.	Shalimov(2013)	Fig. 7			
Goniophlebium chinense (Christ) X.C. Zhang	Mainland China: Sichuan; S.G.Lu 31809 (PYU)	Zhang et al. (2006)	Plate I 9-10	0	0	20–39 × 36–59
	Mainland China: Guizhou; Q.W.Sun 10042818 (HGAS)	Jiang et al. (2010)	Plate I 8			
Goniophlebium formosanum (Baker) Rodl-Linder	Taiwan: Yilan; CC.Chen 016 (SYSU)	This study	Fig.1 C3.1-C3.2	0	0	24–38 × 43–60
	Formosa (Taiwan); Faurie 8428	Tryon & Lugardon (1991)	Figs. 131.4			

	Japan: Yakushima; TNS 321728	Mitui(1977)	Plates C7, D7			
Goniophlebium manmeiense (Christ) Rodl-Linder	Vietnam: Cao Bo; Harder et al., No. 5664	Shalimov(2013)	Fig. 6	0	0	20–30 × 35–46
---------	Mainland China: Guizhou; F.Wang 90183 (HGAS)	Wang (2001)	Plate CII 1-2			
Goniophlebium mengtzeense (Christ) Rodl-Linder	N. & S. Vietnam; No. NTH 2893 & VH 1565	Shalimov(2013)	Fig. 8	2	0	18.5–35 × 34–60
	Mainland China: Guangxi; H.G.Zhou & H.Li 1204 (HGAS)	Wang (2001)	Plate CII 4-5			
Goniophlebium microrhizoma (C.B. Clarke ex Baker) Bedd.	Mainland China: Yunnan; S.G.Lu & C.D.Xu 31807 (PYU)	Zhang et al. (2006)	Plate I 13-14	0	0	17–30 × 36–50
	-	Rödl-Linder, G. (1990)	-			
Goniophlebium niponicum (Mett.) Bedd. var. *niponicum*	Taiwan: Taoyuan; CC.Chen 159 (SYSU)	This study	Fig.1 C4.1-C4.2	0	0	24–45 × 46.5–65
	Mainland China: Kouy-Tcheou; No 2031, Bodinier R.P.	Shalimov(2013)	Fig. 3			
	Mainland China: Zhejiang; Q.X.Wang 090926011 (SHNU)	Jiang et al. (2010)	Plate I 5			
	-	Rödl-Linder, G. (1990)	-			
Goniophlebium niponicum var. *wattii* (Bedd.) Bedd.	Mainland China: Biluo Xueshan, Yunnan; S.G.Lu 31801 (PYU)	Zhang et al. (2006)	Plate I 15-16	0	0	22.5–45 × 48–65
Goniophlebium persicifolium (Desv.) Bedd.	Malaya; Molesworth-Allen 3484	Tryon & Lugardon (1991)	Figs. 132.6	2	0	20–27 × 35–50.5
	Vietnam: Thua Thien-Hue; HAL 10833	Shalimov et al. (2013)	Fig. 9			
Goniophlebium pseudoconnatum (Copel.) Copel.	-	Rödl-Linder (1990)	-	2	0	25 × 40–45
Goniophlebium subauriculatum (Blume) C.Presl	86GR00010 greenhouse Utrecht	Rödl-Linder(1990)	Plate 3:b	2	0	20–35 × 35–50
	Philippines; Bartsch501	Tryon & Lugardon (1991)	Figs. 132.5			
	Mainland China: Yunnan; No. 13145, Henry A.	Shalimov et al. (2013)	Fig. 10			
Lecanopteris carnosa (Reinw.) Blume	New Guinea; Van Royen & Sleumer 6346	Tryon & Lugardon (1991)	Figs. 118.7-8	8	0	33 × 58
	Malaysia; Burkill 740	Tryon & Lugardon (1991)	Figs. 118.9-10			
Lecanopteris celebica Hennipman	Hennipman 5993/5665 (L)	van Uffelen(1993)	Plate III:2&5	8&7	0	33 × 60
Lecanopteris mirabilis (C. Chr.) Copel.	New Guinea; Wormersley 24942	Tryon & Lugardon (1991)	Figs. 118.17-20	9	0	35–75 (genus)
	UBG ex Jongkind s.n., U	Hennipman (1990)	Fig. 2.7:g			
Lecanopteris sinuosa (Hook.) Copel.	Indonesia; Kuswata & Soopadamo 147	Tryon & Lugardon (1991)	Figs. 118.1	7	0	32–38 × 42–48
	New Guinea; Van Royen 4644	Tryon & Lugardon (1991)	Figs. 118.2-5			
Lemmaphyllum carnosum (Wall. ex J. Sm.) C. Presl	DeVol 2398 (MICH)	Hennipman (1990)	Fig. 2.3:d	5	0	26.5–48 × 43–74

Lemmaphyllum drymoglossoides (Baker) Ching	Mainland China: Kwangsi; Steward & Cheo 339	Tryon & Lugardon (1991)	Figs. 113.1	5	0	38–42 × 50–66
	Mainland China: Guizhou; P.S.Wang & W.M.Chu 77553 (HGAS)	Wang (2001)	Plate CIV 1-3			
Lemmaphyllum microphyllum C. Presl	Taiwan: Taipei; CC.Chen 002 (SYSU)	This study	Fig.1 D1.1-D1.2	5	0	30–77.5 × 40–102.5
	Hong Kong; Hu 9403	Tryon & Lugardon (1991)	Figs. 113.2-3			
	Mainland China: Shenzhen; S.F.Wu 20090115-02 (SHNU)	Jiang et al. (2010)	Plate III 10-11			
Lemmaphyllum rostratum (Bedd.) Tagawa	Taiwan: Pingtung; CC.Chen 014 (SYSU)	This study	Fig.1 D2.1-D2.2	5	0	25–51 × 39–65
	Mainland China: Hainan; Lau 3144	Tryon & Lugardon (1991)	Figs. 113.4			
Lepidomicrosorium buergerianum (Miq.) Ching & K.H. Shing	Japan: Okinawa; TNS 321721	Mitui (1977)	Plates B:1; D:1	5	0	25–60 × 34–74
	Mainland China: Zhejiang; X.L.Dai 09110101 (SHNU)	Jiang et al. (2010)	Plate IV 16			
Lepidomicrosorium superficiale (Blume) L. Wang	Taiwan: Pingtung; CC.Chen 008 (SYSU)	This study	Fig.1 E1.1-E1.2	5	0	23–45 × 38–75
	Mainland China: Xizang (Tibet); P.C.Tsoong 6194 (PE)	Jiang et al. (2010)	Plate IV 9			
	Mainland China: Xizang (Tibet); B.Q.Zhong 6194 (PE)	Wang (2001)	Plate CVI 5-6			
Lepisorus accedens (Blume) Hosok.	New Guinea; Croft & March 915A	Tryon & Lugardon (1991)	Figs. 113.5	3	0	48 × 64
Lepisorus affinis Ching	Hainan Island; C.L.Tso & N.K.Chun 44067 (PE)	Qi & Zhang (2009)	Fig.7 A,B	5	0	-
Lepisorus angustus Ching	Japan: Okukinu	Mitui (1971)	Plate XVIII: C,D	5	0	34–45 × 51–67
Lepisorus annuifrons (Makino) Ching	Japan: Kawamata	Mitui (1971)	Plate XIX: A,B	5	0	27.5–47.5 × 47.5–77.5
Lepisorus asterolepis (Baker) Ching ex S.X. Xu	Mainland China: Chongqing; X.P.Qi 4590	Qi & Zhang (2009)	Fig.6 E,F	5	0	47 × 71
	Mainland China: Guizhou; S.F.Wu 090803002	Jiang et al. (2010)	Plate IV 3-4			
Lepisorus boninensis (Christ) Ching	Japan: Tokyo, Chichijima; TNS 321726	Mitui (1977)	Plates C:1, D:9	5	0	31.8 × 48.7
Lepisorus clathratus (C.B.Clarke) Ching	Taiwan: Hualien; CC.Chen 099 (SYSU)	This study	Fig.1 F1.1-F1.2	5	0	21–60 × 36–93
	Japan: Kawamata	Mitui (1971)	Plate XIX: G,H			
	Japan	Mitui (1977)	Plate E:6			
	Bot. Pith. BSK 88105, BSK 89524, KB 200345	Kholia et al. (2012)	Fig.2 A			
Lepisorus contortus (Christ) Ching	Mainland China: Sichuan; Z.G.Liu 780 (CDBI)	Wang (2001)	Plate CIII 9	5	0	32–48 × 48–67
	Mainland China: Sichuan; Z.G.Liu 780 (CDBI)	Jiang et al. (2010)	Plate III 13			
Lepisorus kawakamii (Hayata) Tagawa	Taiwan: Hualian; T.C. Chen 9862	Qi & Zhang (2009)	Fig.6 G,H	5	0	-
Lepisorus kuchenensis (Y.C. Wu) Ching	Taiwan: Taichung; Imaseki 70500 (TI)	Qi & Zhang (2009)	Fig.6 K,L	5	0	-
Lepisorus loriformis (Wall. ex Mett.) Ching	Bot. Pith. BSK 870951, BSK 910617	Kholia et al. (2012)	Fig.2 G	3&5	0	-

Lepisorus macrosphaerus (Baker) Ching	Mainland China: Yunnan; K.M.Feng 12714 (PE)	Qi & Zhang (2009)	Fig.6 A,B	5	0	40–55 × 59–74
	Mainland China: Sichuan; Zaoyuan Exped. 28443 (CDBI)	Wang (2001)	Plate CIII 3			
Lepisorus marginatus Ching	Mainland China: Sichuan; X.Li 72707 (PE)	Qi & Zhang (2009)	Fig.6 C,D	5	0	40 × 62
	Mainland China: Chengkou, Sichuan; T.L.Dai 104790 (CDBI)	Wang (2001)	Plate CIII 4			
	Mainland China: Kangding, Sichuan; X.X.Kong 6892 (CDBI)	Wang (2001)	Plate CIII 5			
Lepisorus megasorus (C. Chr.) Ching	Taiwan: Hualian; T.C.Chen 12733 (PE)	Qi & Zhang (2009)	Fig.6 I,J	5	0	25–58 × 42–79
Lepisorus miyoshianus (Makino) Fraser-Jenk. & Subh. Chandra	Taiwan: Nantou; TY.Tzi 720 (SYSU)	This study	Fig.1 F2.1-F2.2	5	0	36–70 × 49–87
	Mainland China: Kweichow (Guizhou); Tsang 5915	Tryon & Lugardon (1991)	Figs. 112.3			
Lepisorus morrisonensis (Hayata) H. Itô	India: Kumaon region (Bot. Pith.)	Kholia et al. (2012)	Fig.2 B	3&5	0	36–52 × 51–73
Lepisorus mucronatus (Fée) Li Wang	Java; Palmer & Bryant 89	Tryon & Lugardon (1991)	Figs. 111.1	5	0	35–37 × 52–67
	Australia: Queensland; White 10683	Tryon & Lugardon (1991)	Figs. 111.3			
Lepisorus obscurevenulosus (Hayata) Ching	Taiwan: Pingtung; CC.Chen 350 (SYSU)	This study	Fig.1 F3.1-F3.2	5	0	27–50 × 37–64
Lepisorus oligolepidus (Baker) Ching	Mainland China: Guizhou; G.Q.Gou 13-111 (HGAS)	Jiang et al. (2010)	Plate IV 5	5	0	46–63 × 55–80
Lepisorus onoei (Franch. & Sav.) Ching	Japan: Mt. Hakkoda	Mitui (1971)	Plate XVIII: A-B	3&5	0	39–72.5 × 70–102.5
	Japan; Furuse in 1960	Tryon & Lugardon (1991)	Figs. 114.6			
Lepisorus platyrhynchos (Kunze) Li Wang	Philippines; Topping 1175	Tryon & Lugardon (1991)	Figs. 111.4	5	0	-
Lepisorus pseudonudus Ching	India: Bot. Pith. BSK 871105	Kholia et al. (2012)	Fig.2 I	5	0	30 × 45
Lepisorus pseudoussuriensis Tagawa	Taiwan: Wuling Farm; CC.Chen 054 (SYSU)	This study	Fig.1 F4.1-F4.2	3&5	0	20–40 × 37–55
	Taiwan; Tagawa, Chuang & Kao 2430 (L)	Tryon & Lugardon (1991)	Figs. 114.4-5			
Lepisorus rotundus Ching	Kenya; Zogg & Gassner 317/3 (Z)	Zink (1993)	Fig.3 d	5	0	-
Lepisorus scolopendrium (Ching) Mehra & Bir	India: Kumaon region (Bot. Pith.)	Kholia et al. (2012)	Fig.2 C	3&5	0	-
	Mainland China: Yunnan Maguan; S.K.Wu 61-3578 (PE)	Qi & Zhang (2009)	Fig.7 E,F			
Lepisorus spicatus (L.f.) Li Wang	Ceylon	Erdtman & Sorsa (1971)	-	5	0	37–52 × 58–88
	India: Assam, Cherrapunjee, K. & J. Hills; B.K.Nayar 63451	Nayar & Devi (1964)	Figs. 38&94			
Lepisorus sublinearis (Baker ex Takeda) Ching	-	Kholia et al. (2012)	-	5	0	37–50 × 50–75
	-	Devi (1981)	-			

Lepisorus thunbergianus (Kaulf.) Ching	Taiwan: Taipei; CC.Chen 003 (SYSU)	This study	Fig.1 F5.1-F5.2	5	0	22-52.5 × 32-82.5
	Japan: Mugi (2X) & Iwayadera (4X)	Mitui (1971)	Plate XVII: A-H			
	Mainland China: Sichuan; X.X.Kong 6157 (CDBI)	Wang (2001)	Plate CIII 8			
	Mainland China: Zhejiang	Dai et al. (2006)	Plate II: 19-20			
Lepisorus ussuriensis (Regel & Maack) Ching	Japan; Ung22225	Tryon & Lugardon (1991)	Figs. 114.3	3&5	0	32.5-72.5 × 47.5-107.5
Lepisorus soulieanus (Christ) Ching & S.K. Wu	Mainland China: Sichuan, Kangding; X.X.Kong 6893 (CDBI)	Wang (2001)	Plate CIII 1-2	1	0	40 × 60
Lepisorus waltonii (Ching) S.L. Yu	-	Zhang et al. (2003)	-	1	0	-
	-	Zhang et al. (2013)	-			
Leptochilus axillaris (Cav.) Kaulf.	Java; Buysman158	Tryon & Lugardon (1991)	Figs. 119.5	6&7	2	30-32 × 53-64
	Holttum 26219 (PE)	Wang (2001)	Plate CVIII 9			
Leptochilus cantoniensis (Baker) Ching	L.Zheng1568 (PE)	Jiang et al. (2010)	Plate V 15	6&7	2	35 × 45
Leptochilus decurrens Blume	Taiwan: Nantou; Y.C.Liou 0047 (SYSU)	This study	Fig.1 G1.1-G1.2	6&7	2	21-35 × 42-62
	India; Kostermans 26187 (US)	Tryon & Lugardon (1991)	Figs. 119.1-2			
	Mainland China: Guizhou; Y.P.Wang et al. s. n. (GBG)	Jiang et al. (2010)	Plate V 14			
	China: Guizhou; Y.P.Wang et al. s. n. (GBG)	Wang (2001)	Plate CVIII 1-3			
Leptochilus digitatus (Baker) Noot.	Mainland China: Guangdong; L.Shi 97010 (PE)	Shi & Zhang (1998)	Fig. 34-36	6&7	2	22.5-47.5 × 32.5-52.5
Leptochilus ellipticus (Thunb. ex Murray) Noot.	Mainland China: Jiangsu; L.Shi 97015A (PE)	Shi & Zhang (1998)	Fig. 19-21	6&7	2	21-47.5 × 34-72.5
Leptochilus ellipticus var. *flexilobus* (Christ) X.C. Zhang	Mainland China: Yunnan; Tsai 60353	Tryon & Lugardon (1991)	Figs. 116.1	6&7	2	18.8-25 × 32.5-52.5
	Mainland China: Sichuan; K.H.Shing et al. 5195 (PE)	Shi & Zhang (1998)	Fig. 25-27			
Leptochilus ellipticus var. *pentaphyllus* (Baker) X.C. Zhang & Noot.	Mainland China: Yunnan; W.M.Chu et al. 12578 (PYU)/Y.H.Li 5881 (KUN)	Shi & Zhang (1998)	Fig. 28-30	6&7	2	19-29 × 35-59
Leptochilus hemionitideus (C. Presl) Noot.	Taiwan: Ilan FuShan; Liou 2521 (SYSU)	This study	Fig.1 G2.1-G2.2	6&7	2	21-39 × 37-60
	Mainland China: Xizang(Tibet); W.L.Chen 14298 (PE)	Shi & Zhang (1998)	Fig. 1-3			
	Mainland China: Yunnan Maguan; Z.R.Wang 812 (PE)	Wang (2001)	Plate CVIII 7-8			
	Hennipman 3388 (L)	Hennipman (1990)	Fig. 2.1:a			
Leptochilus x *hemitomus* (Hance) Noot.	Cultivated; L.Shi 98057/ China: Guangxi; Z.R.Wang 5209 (PE)	Shi & Zhang (1998)	Fig. 31-33	6&7	2	21-30 × 35-50
	Mainland China: Fujian; J.G.Cao 609095 (SHNU)	Jiang et al. (2010)	Plate V 9			
Leptochilus henryi (Baker) X.C. Zhang	Mainland China: Sichuna; Fang 2383	Tryon & Lugardon (1991)	Figs. 116.2	6&7	2	19-40 × 35-49
	Mainland China: Sichuan, Mt. Emei; K.H.Shing et al. 957 (PE)	Shi & Zhang (1998)	Figs. 13-15			
Leptochilus heterophyllus (S.K. Wu & K.L. Phan) Christenh.	Vietnam: Kon Tum; Wu et al. WP-135	Sugong et al. (2005)	Fig. 2	6&7	2	× 45-65

Leptochilus leveillei (Christ) X.C. Zhang & Noot.	Mainland China: Guangxi; X.C.Zhang 1079 (PE)	Shi & Zhang (1998)	Figs. 16-18	6&7	2	19-29 × 40-49
	Mainland China: Guizhou; F.Wang 165 (HGAS)	Wang (2001)	Plate CVIII 6			
	Mainland China: Guizhou; F.Wang 165 (HGAS)	Jiang et al. (2010)	Plate V 13			
Leptochilus macrophyllus (Blume) Noot.	Philippines; Jacobs 755	Tryon & Lugardon (1991)	Figs. 116.3-4	6&7	2	34 × 73
Leptochilus pedunculatus (Hook. & Grev.) Fraser-Jenk.	Mainland China: Yunnan; W.M.Chu 2085 (PYU)/Kunming Inst. Eco. (PE)	Shi & Zhang (1998)	Figs. 7-9	6&7	2	25-37.5 × 40.5-54
	Malaya; Molesworth-Allen 4132	Tryon & Lugardon (1991)	Figs. 116.7-8			
Leptochilus pothifolius (Buch.-Ham. ex D. Don) Fraser-Jenk.	Mainland China: Guangdong; L.Shi 97039 (PE)	Shi & Zhang (1998)	Figs. 22-24	6&7	2	22.5-35 × 37.5-55
	Mainland China: Fujian; J.G.Cao 609030 (SHNU)	Jiang et al. (2010)	Plate V 12			
Leptochilus pteropus (Blume) Fraser-Jenk.	Taiwan: Mt. Mopitan; Pi-Fong Lu 29763 (TAIF 496040)	This study	Fig.1 G3.1-G3.2	6&7	2	20-35 × 40-59
	s.n. 1707 (PE)	Wang (2001)	Plate CVII 13-14			
Leptochilus wrightii (Hook. & Baker) X.C. Zhang	Taiwan: Bolu Shan; C.C.Chen 118 (SYSU)	This study	Fig.1 G4.1	6&7	2	17.5-45 × 33-81
	Taiwan: Pingtung; C.C.Chen 190 (SYSU)	This study	Fig.1 G4.2			
	Hong Kong; T.J.Li 001/ China:Yunnan; Yunnan Univ. 1017(PE)	Shi & Zhang (1998)	Figs. 10-12			
Microsorum commutatum (Bl.) Copel.	82GR00146 (HBU)	Van Uffelen (1993)	Plate IV:8	6&7	1	31 × 45-65
	LBG 21238 (L)	Hennipman(1990)	Fig. 2.1:e			
Microsorum cuspidatum (D. Don) Tagawa	Nepal: Kaski District; C.R. Fraser-Jenkins, FN287 (TAIF 310152)	This study	Fig.1 H1.1-H1.2	7	0	34-57 × 42-84
	Mainland China: Guizhou; F.Wang 177 (HGAS)	Jiang et al. (2010)	Plate II 3-4			
Microsorum glossophyllum Copel.	-	Petchsri et al. (2012)	-	1	0	20-45 × 50-80
	-	Bosman (1991)	-			
Microsorum hainanense Noot.	Hainan Island; K.S.Chow 78461	Shi (2002)	Plate I:8-9	7	0	36-61.5 × 56-94.5
	Hainan Island; S.F.Wu 09007 (SHNU)	Jiang et al. (2010)	Plate II 5-6			
	K.S.Chow 78707 (PE)	Wang (2001)	Plate CVII:4-5			
Microsorum insigne (Blume) Copel.	Taiwan: Pingtung, Mt. Maotzu; P.F.Lu 27122 (TAIF 454246)	This study	Fig.1 H2.1-H2.2	7	0	20-35 × 34-70
	Z.Y.Zhang 64-43 (PE)	Wang (2001)	Plate CVII:9-10			
Microsorum membranifolium (R. Br.) Ching	Ceylon; Davidse 8359	Tryon & Lugardon (1991)	Figs. 122.6	6&7	1	25-44 × 41-77
	India; Kramer & Nair 6153	Tryon & Lugardon (1991)	Figs. 122.7-8			
	K.M.Feng 4687 (PE)	Wang (2001)	Plate CVII:1-2			
	China: Yunnan; K.M.Feng 4687	Shi (2002)	Plate I:7			
Microsorum musifolium (Blume) Copel.	Papua; Brass 6602	Tryon & Lugardon (1991)	Figs. 120.16	1	0	24-40 × 37-55

Microsorum punctatum (L.) Copel.	Taiwan: Pingtung; CC.Chen 149 (SYSU)	This study	Fig.1 H3.1-H3.2	1	0	20-49 × 36-75
	Ceylon; Beckett 648	Tryon & Lugardon (1991)	Figs. 120.1-2			
	Madagascar; Croat 30124	Tryon & Lugardon (1991)	Figs. 120.3			
	Siam; Den Hoed 56433	Tryon & Lugardon (1991)	Figs. 120.14			
Microsorum rubidum (Kunze) Copel.	Taiwan: Pingtung; CC.Chen 113 (SYSU)	This study	Fig.1 H4.1-H4.2	6&7	1	20-50 × 35-105
	Papua; Brass 27124	Tryon & Lugardon (1991)	Figs. 122.9-10			
	Mainland China: Hainan; Tso&Chun 43820	Shi (2002)	Plate I:4			
	Amdjah129 (L)	Hennipman(1990)	Fig. 2.4:f-g			
Microsorum scolopendria (Burm. f.) Copel.	Solomon: Ringgi Village; SITW02007 (TAIF 442937)	This study	Fig.1 H5.1	5	0	23-50 × 34-76.5
	Taiwan: Tainan; CC.Chen 103 (SYSU)	This study	Fig.1 H5.2			
	Japan: Okinawa, Iriomote; TNS 321722	Mitui (1977)	Plate B:5 & D:8			
	Sumatra; korthals s.n.	Tryon & Lugardon (1991)	Figs. 122.1			
	Cameroons; Tryon&Tryon 6496	Tryon & Lugardon (1991)	Figs. 122.2			
	New Guinea; Brass 13738	Tryon & Lugardon (1991)	Figs. 122.3-4			
	H.Y.Liang 62110 (PE)	Wang (2001)	Plate CVI:13-14			
	-	van Uffelen (1993)	-			
Microsorum steerei (Harr.) Ching	Taiwan: Taichung, Lileng; H.L. Chiang 2963 (TAIF 175258)	This study	Fig.1 H6.1-H6.2	1	0	24-44 × 38-70
	-	Petchsri et al. (2012)	-			
	-	Bosman (1991)	-			
Microsorum thailandicum T. Booknerd & Noot.	Taiwan: KBCC; Y.L. Chang K013591, K013594 (TAIF 508278)	This study	Fig.1 H7.1-H7.2	1	0	41-61 × 64-86
	-	Petchsri et al. (2012)	-			
Neocheiropteris palmatopedata (Baker) Christ	Mainland China: Lou Pau; Maire	Tryon & Lugardon (1991)	Figs. 121.3	1	0	29-38 × 44-54
	Mainland China: Guizhou; Y.P.Wang et al. s.n. (GBG)	Wang (2001)	Plate CII:6-7			
Neolepisorus ensatus (Thunb.) Ching	Taiwan: South Cross-lsland Highway; Y.N.Co 0393 (SYSU)	This study	Fig.1 I1.1-I1.2	5	0	29-52.5 × 45-82.5
	Japan; Ando 70312 & Siebold in 1829 (L)	Tryon & Lugardon (1991)	Figs. 121.1-2			
	Mainland China: Sichuan; s. coll. 4053 (PE)	Wang (2001)	Plate CVI:7-8			
	Mainland China: Sichuan; Anonymous 4053 (PE)	Jiang et al. (2010)	Plate IV 12			
Neolepisorus fortunei (T.Moore) Li Wang	Taiwan: Central Cross-lsland Highway; CC.Chen 049 (SYSU)	This study	Fig.1 I2.1-I2.2	5	0	22-45 × 37-74
	Qinghai-Xizang Exped. 73-861 (PE)	Wang (2001)	Plate CVI:1-2			

Neolepisorus ovatus (Wall. ex Bedd.) Ching	Mainland China: Guizhou; Q.X.Wang et al. s.n. (SHNU)	Jiang et al. (2010)	Plate III 8	5	0	20-35 × 28-50
	Esquirol 1105 (P)	van Uffelen(1993)	Plate III 8			
Neolepisorus zippelii (Blume) L. Wang	Indonesia: Lombok; Elbert 2454	Tryon & Lugardon (1991)	Figs. 120.7-9	5	0	25-40 × 45-64
	Sino-Russia Exped. 927 (PE)	Wang (2001)	Plate CVI:3-4			
Paragramma longifolia (Blume) T. Moore	LBG 123306 (L)	Hennipman (1990)	Fig. 2.3:f	5	0	35-41 × 50-66
	Malaya; Lee & Ul 48 (L)	Tryon & Lugardon (1991)	Figs. 114.1			
Thylacopteris papillosa (Blume) J.Sm.	Jahansson c.s. 84	Rödl-Linder (1994)	Fig. 3:c-d	7	0	42 × 54-66
Tricholepidium normale (D. Don) Ching	India: Meghalaya; C.R. Fraser-Jenkins, FN268 (TAIF 319710)	This study	Fig.1 J1.1-J1.2	5	0	32-45 × 38-67
	Mainland China: Yunnan; Tsai 56433	Tryon & Lugardon (1991)	Figs. 120.6, 120.15			
	India: Assam, Shillong, Ward Lake; B.K.Nayar 49807	Nayar & Devi (1964)	Figs. 95 (LM)			
Zealandia novae-zealandiae (Baker) Testo&A. R. Field	New Zealand: Mt. Ruapehu; M.F.Large AKU 22119	Large et al. (1992)	Fig. 1:C	0	0	32-44 × 51-70
	New Zealand: Erua, Tongariro; M.F.Large MFL154 (AKU 22119)	Large & Braggins (1991)	Fig. 224			
Zealandia pustulata (G. Forst.) Testo&A. R. Field	New Zealand; G.Forster (BM)	Large et al. (1992)	Fig. 1:A	0	0	14-40 × 31-60
	New Zealand: Auckland; M.F.Large MFL153b (AKU 22118)	Large & Braggins (1991)	Fig. 221			
Zealandia powellii (Baker) Testo & A. R. Field	Solomon: Kolombangara; C.W.Chen et al. SITW04893 (TAIF)	This study	Fig.1 K1.1-K1.2	1	0	30-39 × 51-62
Aglaomorpha meyeniana Schott	Taiwan: Nanjenshan Nature Reserve, CC.Chen 222 (SYSU)	This study	Fig.1 L1.1-L1.2	0	3	25-42 × 41-65
	Philippines; Topping 802	Tryon & Lugardon (1991)	Figs. 109.10-109.11			
	Price 1049 (MICH)	Hennipman(1990)	Fig. 2.5:h			
Pyrrosia polydactyla (Hance) Ching	Taiwan: Central Cross-lsland Highway; CC.Chen 047 (SYSU)	This study	Fig.1 M1.1-M1.2	0	0	38-71 × 55-86
	LEI21249	van Uffelen (1993)	Plate I 3			

Table S2. Mean posterior probabilities of the Bayesian reconstruction for the ancestral states of three studied characters for 22 nodes. The ancestral states with highest posterior values are marked **in bold**. The abbreviation of states indicates: Ver., verrculate; Psi., Psilate; Ver.+crest, Verrucate with longitudinal crest; Tub., Tuberculate; Verm –pap., Vermiculate-papillate; Rug., Rugulate; Spi., Spinose; Glo., Globular; She., sheath-like; Cab., Cable-like ; Abs., Abscent; Short, Shorter spinose; Long, Longer spinose; Bacu., Baculate . The abbreviation a–v corresponds to nodes of the microsoroid ferns. All nodes are marked in Fig. 3a and Fig. 4. The visualization of the data exhibits in Fig. 4.

Character		Surface main ornamentation										Spinose/Baculate			
state		Ver.	Psi.	Ver.+ crest	Tub.	Verm -pap.	Rug.	Spi.	Glo.	she.	Cab.	Abs.	Short	Long	Bacu.
Nodes	a	**0.8077**	0.0045	0.0074	0.0051	0.0192	0.0054	0.005	0.1306	0.0067	0.0085	**0.9991**	0.0003	0.0002	0.0003
	b	**0.9263**	0.0012	0.0045	0.0009	0.0197	0.004	0.0008	0.0388	0.001	0.003	**0.9999**	0.0000	0.0000	0.0001
	c	**0.8649**	0.0002	0.133	0.0003	0.0004	0.0001	0.0003	0.0001	0.0003	0.0004	**1.0000**	0.0000	0.0000	0.0000
	d	**0.6496**	0.0082	0.0048	0.0067	0.1171	0.0437	0.0054	0.1451	0.0074	0.012	**0.9986**	0.0001	0.0000	0.0014
	e	**0.6348**	0.0036	0.0054	0.0058	0.3125	0.0017	0.0049	0.0154	0.0065	0.0094	**0.8535**	0.0027	0.0009	0.1429
	f	**0.9536**	0.0004	0.0006	0.0006	0.0006	0.0002	0.0006	0.03	0.0053	0.008	**1.0000**	0.0000	0.0000	0.0000
	g	**0.8197**	0.0039	0.005	0.0051	0.0053	0.0024	0.0049	0.1393	0.0069	0.0074	**0.9996**	0.0001	0.0001	0.0001
	h	0.0220	0.0052	0.0045	0.0077	0.0058	0.0024	0.0077	0.2706	0.2633	**0.4105**	**0.9999**	0.0000	0.0000	0.0000
	i	0.0672	0.0447	0.0136	0.0211	0.0161	0.2607	0.0203	**0.5086**	0.023	0.0245	**0.9994**	0.0004	0.0001	0.0001
	j	0.0007	0.0099	0.0001	0.0003	0.0003	0.0012	0.003	**0.9838**	0.0002	0.0003	**0.9874**	0.0110	0.0015	0.0001
	k	0.0036	0.1069	0.0012	0.0029	0.0021	0.0085	0.0163	**0.8526**	0.0028	0.0031	**0.9943**	0.0055	0.0001	0.0001
	l	0.0011	**0.9316**	0.0006	0.0011	0.0008	0.0608	0.0007	0.0014	0.0008	0.0011	**0.9999**	0.0000	0.0000	0.0000
	m	0.001	0.0005	0.0005	0.0006	0.0006	0.0003	0.0314	**0.9636**	0.0008	0.0008	**0.6509**	0.1344	0.2095	0.0052
	n	0.001	0.0005	0.0005	0.0006	0.0006	0.0003	0.0143	**0.9806**	0.0008	0.0008	**0.7097**	0.2854	0.0020	0.0030
	o	0.001	0.0006	0.0005	0.0007	0.0007	0.0003	0.4730	**0.5215**	0.0009	0.0008	0.0002	0.0001	**0.9996**	0.0001
	p	0.0004	0.0004	0.0002	0.0003	0.0002	**0.9975**	0.0002	0.0002	0.0002	0.0003	**0.9998**	0.0001	0.0001	0.0001
	q	0.0002	0.0003	0.0001	0.0013	0.0001	**0.9976**	0.0001	0.0001	0.0001	0.0001	**0.9999**	0.0000	0.0000	0.0000
	r	0.0018	0.0022	0.0012	0.0524	0.0014	**0.9356**	0.0014	0.001	0.0015	0.0015	**0.9999**	0.0000	0.0000	0.0000
	s	0.0001	0.002	0.0001	0.0001	0.0001	**0.9973**	0.0001	0.0001	0.0001	0.0001	**0.9999**	0.0000	0.0000	0.0000
	t	0.0000	0.0004	0.0000	0.0000	0.0000	**0.9995**	0.0000	0.0000	0.0000	0.0000	**1.0000**	0.0000	0.0000	0.0000
	u	0.0001	0.0113	0.0001	0.0001	0.0001	**0.9881**	0.0001	0.0001	0.0001	0.0001	**1.0000**	0.0000	0.0000	0.0000
	v	0.0003	0.0003	0.0002	0.0002	0.0002	**0.9982**	0.0002	0.0002	0.0002	0.0002	**0.9998**	0.0001	0.0000	0.0001
